# Power analyses for measurement model misspecification and response shift detection with structural equation modeling

**DOI:** 10.1007/s11136-024-03605-3

**Published:** 2024-03-01

**Authors:** M. G. E. Verdam

**Affiliations:** 1 Department of Methodology and Statistics, Institute of Psychology, Leiden University, P.O. Box 9555, 2300 RB Leiden, The Netherlands; 2 Medical Psychology, Amsterdam UMC Location University of Amsterdam, Meibergdreef 9, Amsterdam, The Netherlands

**Keywords:** Statistical power, Sample size planning, Structural equation modeling, Response shift, Chi-square test, Root mean square error of approximation (RMSEA)

## Abstract

**Purpose:**

Statistical power for response shift detection with structural equation modeling (SEM) is currently underreported. The present paper addresses this issue by providing worked-out examples and syntaxes of power calculations relevant for the statistical tests associated with the SEM approach for response shift detection.

**Methods:**

Power calculations and related sample-size requirements are illustrated for two modelling goals: (1) to detect misspecification in the measurement model, and (2) to detect response shift. Power analyses for hypotheses regarding (exact) overall model fit and the presence of response shift are demonstrated in a step-by-step manner. The freely available and user-friendly R-package lavaan and shiny-app ‘power4SEM’ are used for the calculations.

**Results:**

Using the SF-36 as an example, we illustrate the specification of null-hypothesis (H_0_) and alternative hypothesis (H_1_) models to calculate chi-square based power for the test on overall model fit, the omnibus test on response shift, and the specific test on response shift. For example, we show that a sample size of 506 is needed to reject an incorrectly specified measurement model, when the actual model has two-medium sized cross loadings. We also illustrate power calculation based on the RMSEA index for approximate fit, where H_0_ and H_1_ are defined in terms of RMSEA-values.

**Conclusion:**

By providing accessible resources to perform power analyses and emphasizing the different power analyses associated with different modeling goals, we hope to facilitate the uptake of power analyses for response shift detection with SEM and thereby enhance the stringency of response shift research.

**Supplementary Information:**

The online version contains supplementary material available at 10.1007/s11136-024-03605-3.

## Introduction

Interpretation of change in self-reports is difficult when it is affected by a change in the meaning of respondents’ self-evaluation, also known as response shift [1]. Response shift research has received increasing attention over the last decades, which has resulted in both theoretical and methodological advances (e.g., see the recent Response Shift—in Sync Working Group initiative [2–5]. Structural equation modeling (SEM) is currently the most widely used statistical approach for the investigation of response shift [6]. When investigating the presence of response shift using statistical hypothesis testing it is important to also consider the statistical power of the test. That is, one needs to consider the chance that the statistical test will be able to detect the response shift effect of interest when this effect truly exists. Low statistical power indicates that even if the response shift effect exists in reality, there is only a small chance that the statistical test will be able to detect it. In order to prevent allocating valuable resources to research with low statistical power, it is thus of utmost importance to consider a-priori power calculations (e.g., [7]).

The power of a statistical test depends on the size of the sample (N), the significance criterion (α), and the effect-size (ES) of the effect of interest in the population. A-priori power calculations are generally performed to be informed about the minimal required sample size to achieve sufficient statistical power. Because the significance criterion and the desired statistical power are usually set at 0.05 and 0.80 respectively, the calculation requires ‘only’ the specification of the population effect-size. However, determining this effect-size is not straightforward. With SEM analyses this is especially complicated because the effect-size that needs to be specified depends on many parameters in the model. Therefore, instead, one often relies on general rules of thumb about sample size (e.g. > 100 or > 200 [8, 9]) or sample size in relation to the number of parameters or variables in the model (e.g. [10, 11]). However, these rules of thumb are problematic because they are not model- or hypothesis-specific and may thus lead to over- or under-estimation of the required sample size; and consequently, to over- or under-powered studies.

The importance of statistical power and the resulting sample-size requirements for response shift detection methods have been emphasized in the literature (e.g. [4, 12]), but in practice power calculations are rarely reported [6]. In part, this may be due to the general complexity of effect-size calculations for SEM analyses. Another complication with power calculations for the detection of response shift is that the SEM approach includes two distinct modeling goals. One modeling goal is that the model as a whole describes the data well; another modeling goal is to test significance of (or differences between) specific model parameters, that is, the response shift effects. The first goal requires that an analysis has enough power to detect a meaningful level of model misspecification; the second goal requires that an analysis has enough power to detect a minimally meaningful effect-size corresponding to a specific parameter (i.e., response shift effect).

Therefore, the aim of the current paper is to provide accessible examples of power-calculations that are relevant for the two modelling goals that are part of the response shift detection approach with SEM; that is, (1) power to detect misspecification in the measurement model (i.e., the test of overall model fit), and (2) power to detect response shift. The latter power calculation can be applied to the overall test for response shift, but also for the detection of individual cases of response shift. Although there exists a number of excellent general tutorial papers on power calculations with SEM (e.g., [13–15]), their uptake in the research area of response shift may be limited due to their general scope and relatively technical language. The original paper of the SEM approach for response shift detection does describe power-calculations for SEM [16], but without the syntaxes being available these calculations may be hard to follow. In the current paper, technical formulas and language are avoided as much as possible to maximize readability for a general audience, but some basic knowledge of Oort’s SEM method [16] is desirable. The recently developed user-friendly and freely available shiny-app ‘power4SEM’ [13] will be used to further facilitate application of power-calculations in practice. Describing power-calculations for response shift detection with SEM in more detail also enables us to emphasize that one needs to consider different power-calculations for the different steps in the modelling procedure. In doing so, we hope to aid researchers with an interest in applying SEM for the detection of response shift in both understanding and using power-calculations, thereby enhancing the stringency of response shift research. Some knowledge on the general notions of statistical power [17, 18] and response shift [1, 3] may benefit those readers who are new to these topics. Some familiarity with the SEM approach for response shift detection [16] is recommended.

## Power calculations

### Illustrative example

**Fig. 1:**
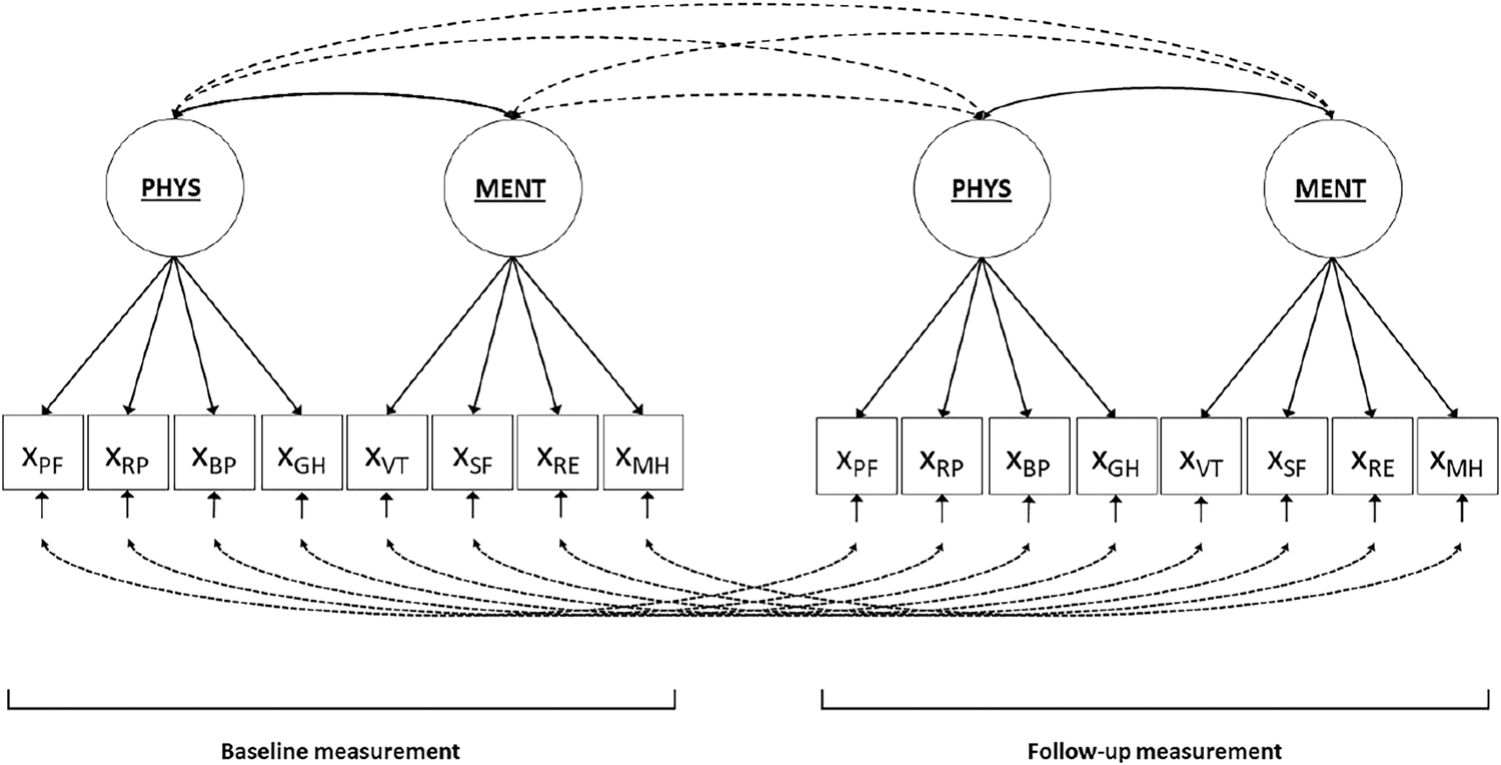
Measurement model of health-related quality of life as measured with the SF-36. *Notes*: Circles at the top represent underlying latent variables general physical health (PHYS) and general mental health (MENT). The squares represent the observed variables (X), i.e., the subscale scores of the SF-36: physical functioning (PF); role physical (RP); bodily pain (BP); general health (GH); vitality (VT); social functioning (SF); role emotional (RE); mental health (MH). The single-headed arrows from the latent variables to the observed variables represent factor loadings. The double headed arrows between the underlying latent variables represent correlations between general physical and general mental health; the underlying latent variables are allowed to correlate over time (dotted double headed arrows). The single headed arrows at the bottom represent residual factors, where each residual factor is associated with one observed variable and only the residual factors of the same variable are allowed to correlate over time (dotted double headed arrows)

To illustrate power-calculations for response shift detection with SEM, we use—following [16]—the SF-36 health-related quality of life questionnaire as an example ([19]; see Fig. 1). That is, the eight subscales of the SF-36 are modelled to be indicative of two underlying latent factors: general physical health and general mental health; measured at two occasions. As questionnaires from the SF-family are most often used in response shift research [20], using the SF-36 as an example is believed to be intuitive for anyone interested in the investigation of response shift.

Appendices I–III include the lavaan syntax specification [21] of all models that are used for the chi-square based power calculations of the SEM approach for response shift that are described below, including descriptive details on the model specification and model parameter values.

### Step 1: Chi-square based power to detect misspecification of the measurement model

The first step of the SEM approach for the detection of response shift entails the specification of the measurement model. This measurement model specifies the measurement structure of the data, where the scores on the observed variables (e.g., scores on questionnaire items or, in this case, scores on the subscales of the SF-36) are related to one or more underlying latent variables (e.g., general mental and physical health) (see Fig. 1). A correctly specified measurement model is important because the measurement model serves as a comparison for all subsequent models. When the measurement model is not correctly specified (e.g., the number of underlying factors is wrong, or the observed variables are related to another underlying latent variable), this will likely affect subsequent results with regards to detection of response shift effects [4]. Therefore, it is important to calculate the statistical power to detect possible misspecification of the measurement model.

Power calculations depend on the type of statistical test that is used. The model fit of the measurement model is usually evaluated with the chi-square test of exact fit. The null-hypothesis (H_0_) that is evaluated with the chi-square test is that the model fits the data exactly. In our example, the H_0_ represents that the measurement of the SF-36 as depicted in Fig. 1 fits the data exactly (i.e., is the ‘true’ model in the population). When the *p*-value falls below the significance criterion (α), we reject H_0_ in favor of the alternative hypothesis. The alternative hypothesis (H_1_) is that the model does not fit the data exactly. In our example, rejecting H_0_ would indicate that the Fig. 1 measurement model of the SF-36 is not the ‘true’ model in the population. Incorrectly rejecting H_0_ is called a Type I error; which is usually set at a 0.05 value. A type II error (β) is made when H_0_ should have been rejected, but was incorrectly retained. In our example, this would mean that the Fig. 1 measurement model of the SF-36 is not rejected, even though it is not the ‘true’ model in the population. The power of a statistical test is the chance to correctly reject H_0_ (1-β; see Table 1).

**Table 1:** Statistical power for the three tests in steps 1–3 of the SEM approach to detect response shift

Reality statistical test	H_0_ = true	H_1_ = true
Reject H_0_	α (Type I error) Step 1: Incorrectly reject measurement model Step 2: Incorrectly reject no response shift model Step 3: Incorrectly reject no response shift parameter	1-β (Power) Step 1: Correctly reject measurement model Step 2: Correctly reject no response shift model Step 3: Correctly reject no response shift parameter
Not reject H_0_	1-α (Correct inference) Step 1: Correctly retain measurement model Step 2: Correctly retain no response shift model Step 3: Correctly retain no response shift parameter	β (Type II error) Step 1: Fail to reject misspecified measurement model Step 2: Fail to reject no response shift model Step 3: Fail to reject no response shift parameter

*H*_*0*_ null-hypothesis, *H*_*1*_ alternative hypothesis

Power calculations require the specification of H_0_ and H_1_. With a simple statistical test like a student *t*-test, H_0_ is usually zero (e.g., there is no difference between groups) and H_1_ is usually set at an effect-size value that is deemed plausible or minimally relevant (e.g., a mean difference according to rules of thumb of small, medium or large effects). Power calculations for the chi-square test of exact fit are based on the difference in chi-square distributions between H_0_ and H_1_, and therefore require the specification of both H_0_ and H_1_ models [22]. Following Oort [16], the H_0_ model for the SF-36 could be the measurement model as specified in Fig. 1. This model works well as an illustration, because it has simple structure (i.e., each variable loads on only one underlying latent factor) and is therefore relatively easy to specify and interpret. The H_1_ model can be any alternative measurement model of the SF-36. Determining a plausible H_1_ model is complicated because model misspecification generally does not entail a specific effect of interest within the model. There thus exist many different options for the definition of H_1_, e.g., a one-factor model, a three-factor model, or a model with one or multiple cross-loadings. Moreover, the calculation of an effect size for H_1_ requires that the values for *all model parameters* in the H_1_ model are specified. It may thus take quite some deliberation on what the exact misspecification should entail. An approach that one could take is to first specify the model under H_0_, i.e., the model that the researcher thinks is the plausible model, including plausible values for all model parameters. Subsequently, one could think of a variation of the H_0_ model that includes one or more additional parameters for which—if these parameters are not zero—the H_0_ model should be rejected. For example, with regards to the measurement model of the SF-36 from our illustrative example, one could think of an alternative measurement model that includes additional loadings (i.e., cross-loadings) of the indicators GH, VT and/or SF that have been previously described in the SF-36 manual [19]. With regards to the value of these additional parameters, the recommendation would be to choose the minimum value that would be of interest. In general, specifying the values of model parameters in standardized metric is convenient because they can be interpreted according to general rules of thumb for representing small, medium, and large effects. For example, standardized factor loadings of 0.1, 0.3 and 0.5 can be interpreted as correlation coefficients and thus represent small, medium and large respectively [7]. Also, previous findings can be used to inform plausible model parameter values.

#### Specification of H_0_ and H_1_ for the Step 1 chi-square based power calculation

Using the illustrative example, the H_0_ model of the SF-36 is defined as depicted in Fig. 1 (see also Appendix I, page 1). It is based on information of the 8 subscales of the SF-36 at baseline and follow-up. The number of unique elements in the variance-covariances matrix of the empirical data is 16*17/2 = 136. Adding the information about the means of the 8 subscales at baseline and follow-up occasion results in a total of 136 + 16 = 152 information statistics. The H_0_ model contains the specification of 16 factor loadings, 4 underlying latent factor variances, 6 underlying latent factor covariances, 16 residual factor variances, 8 residual factor covariances, 16 intercepts, and 4 underlying latent factor means. Identification of the model requires that either the underlying latent factor variance or one factor loading for each latent factor is restricted to a fixed value [16], and that either the mean of the underlying latent factors or one intercept for each latent factor is restricted to fixed values [16]. The total number of free parameters in the H_0_ model is therefore 62 (see also Appendix I, page 5).

**Fig. 2:**
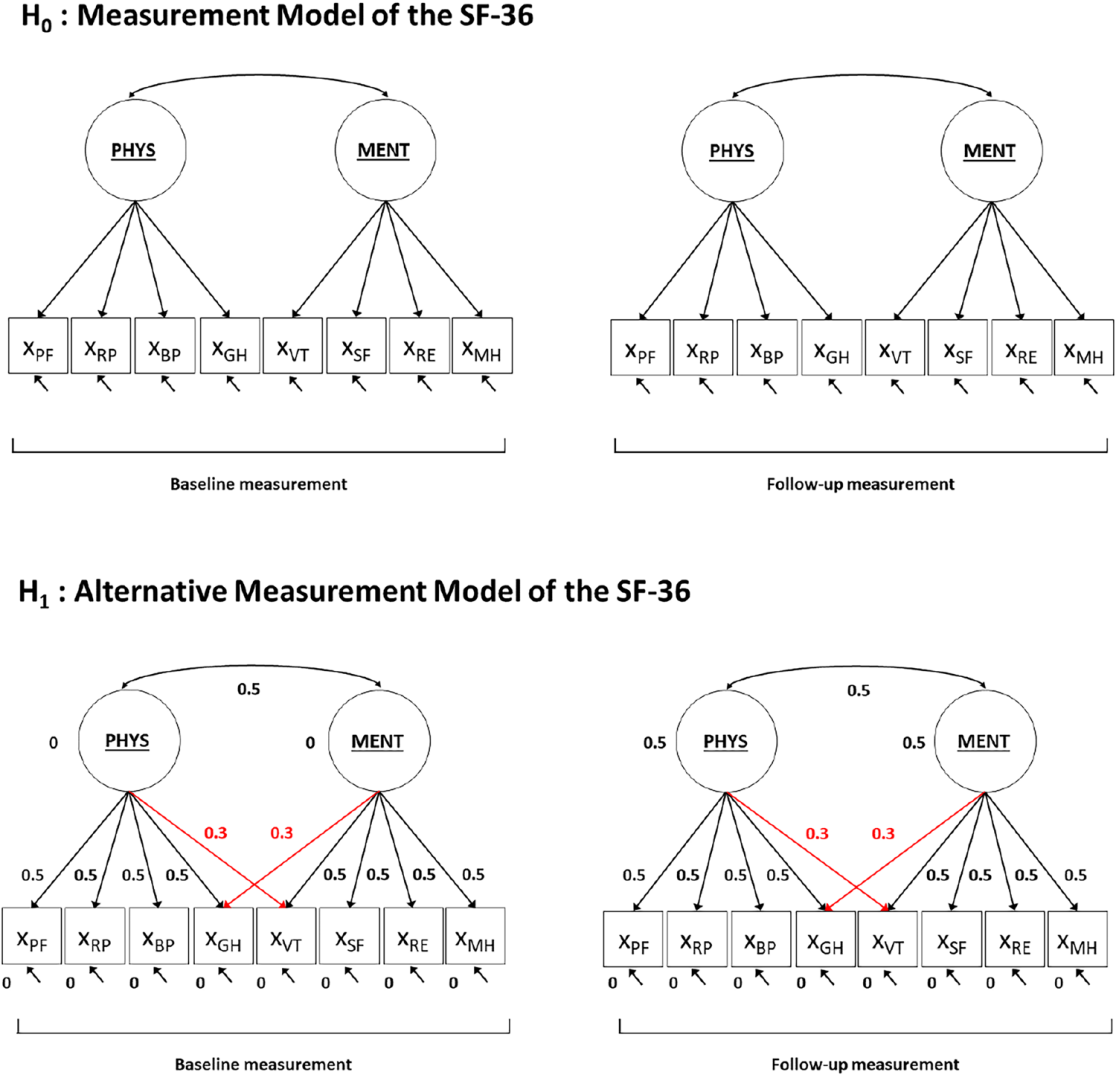
Null-hypothesis (H_0_) model and alternative hypothesis (H_1_) model, including values for the H_1_ model parameter values, used in power calculations for the chi-square test applied in step 1 of the SEM approach for response shift detection. *Notes*: Circles represent underlying latent variables general physical health (PHYS) and general mental health (MENT). The squares represent the observed variables (X), i.e., the subscale scores of the SF-36: physical functioning (PF); role physical (RP); bodily pain (BP); general health (GH); vitality (VT); social functioning (SF); role emotional (RE); mental health (MH). The arrows from the underlying latent variables to the observed variables represent factor loadings, with associated parameter values (for H_1_). The double headed arrow between the underlying latent factors represent the correlation between the latent factors of the same occasion, with associated parameter values (for H_1_). The single-headed arrows at the bottom of each observed variable represent residual factor variances. The values at the bottom of the observed variables (with H_1_) represent intercept values, and the values next to the underlying latent factors (with H_1_) represent latent factor means. The red parameter values refer to the additional parameters present in the H_1_ model that represent the model misspecification, where all model parameters need to be assigned values to be able to calculate an effect-size for the misspecification. Note that the longitudinal relations between underlying latent factors and residual factors of the same observed variable are not depicted here for reasons of conciseness (but see Fig. 1)

The H_1_ model is defined as the H_0_ model with the addition of two medium-sized cross-loadings of the GH and VT subscales (see Fig. 2). Note that there are multiple options for defining H_1_. This specific H_1_ was considered a plausible alternative model based on previous research that has found substantial cross-loadings in the measurement model of the SF-36 (e.g., [23, 24]). We specified the parameter values in standardized form, where the values for the factor loadings are chosen to be 0.5 (i.e., of large size [7]) and the values of the variances of the residual factors are chosen so that the total variance of each observed variable is 1. Similarly, the variances of the underlying latent factors are standardized. This entails that also the values for associations between the residual factors and between the underlying factors can be interpreted as correlation coefficients. The additional cross-loadings in H_1_ were specified to be of medium size (i.e., 0.3 [7]). As choosing parameter values for all parameters in the H_1_ is arguably the most difficult part of chi-square based power calculations, we return to this issue in the discussion section.

#### Step 1 chi-square based power calculation with power4SEM

**Fig. 3:**
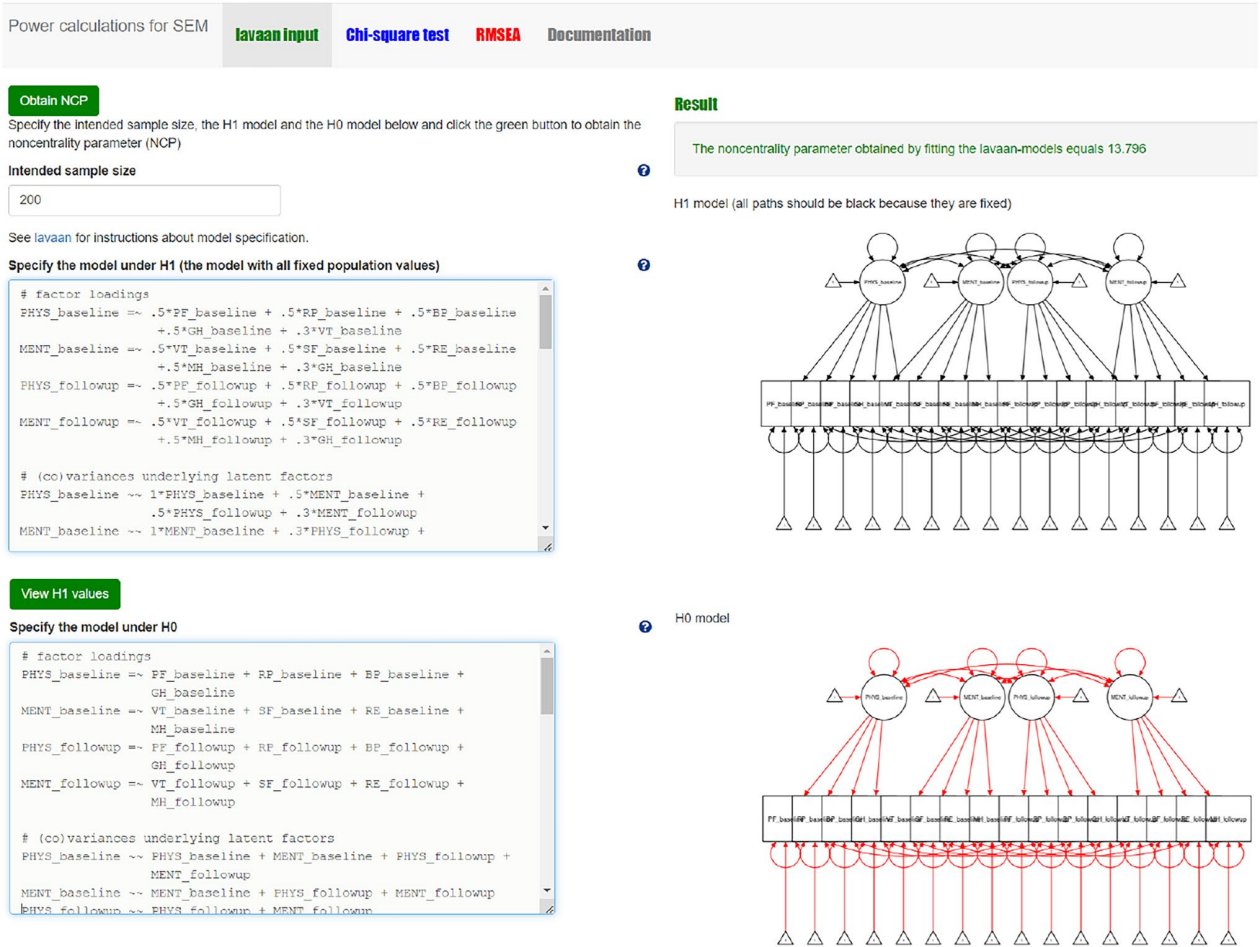
Screenshot of the first step in power calculation for the chi-square test of overall model fit of the measurement model with power4SEM: inserting the H_0_ and H_1_ model syntax

When both models are specified, and plausible values for all model parameters of H_1_ are provided, we can use power4SEM to calculate the chance to correctly reject H_0_. For reasons of conciseness, we will only describe what steps to take in order to arrive at the desired result. We will not go into (technical) details of the underlying calculations or required input values, for which the reader is referred to the tutorial paper of power4SEM [13] and/or the help files available under the question mark buttons on the webpage. In addition, Appendix I also includes a more detailed visual description of the required procedure. As a first step, insert the lavaan syntax of the H_1_ and H_0_ models in the dedicated areas from the “lavaan input” page. You will see a graphical display of both models at the right-hand side of the screen (see Fig. 3). Use the default setting of N = 200 for the “Intended sample size” box; when the researcher has information on the intended or acquired sample size for the proposed/performed study, one could inserted that specific number instead. Click on the green button “obtain NCP” at the top of the page.

**Fig. 4:**
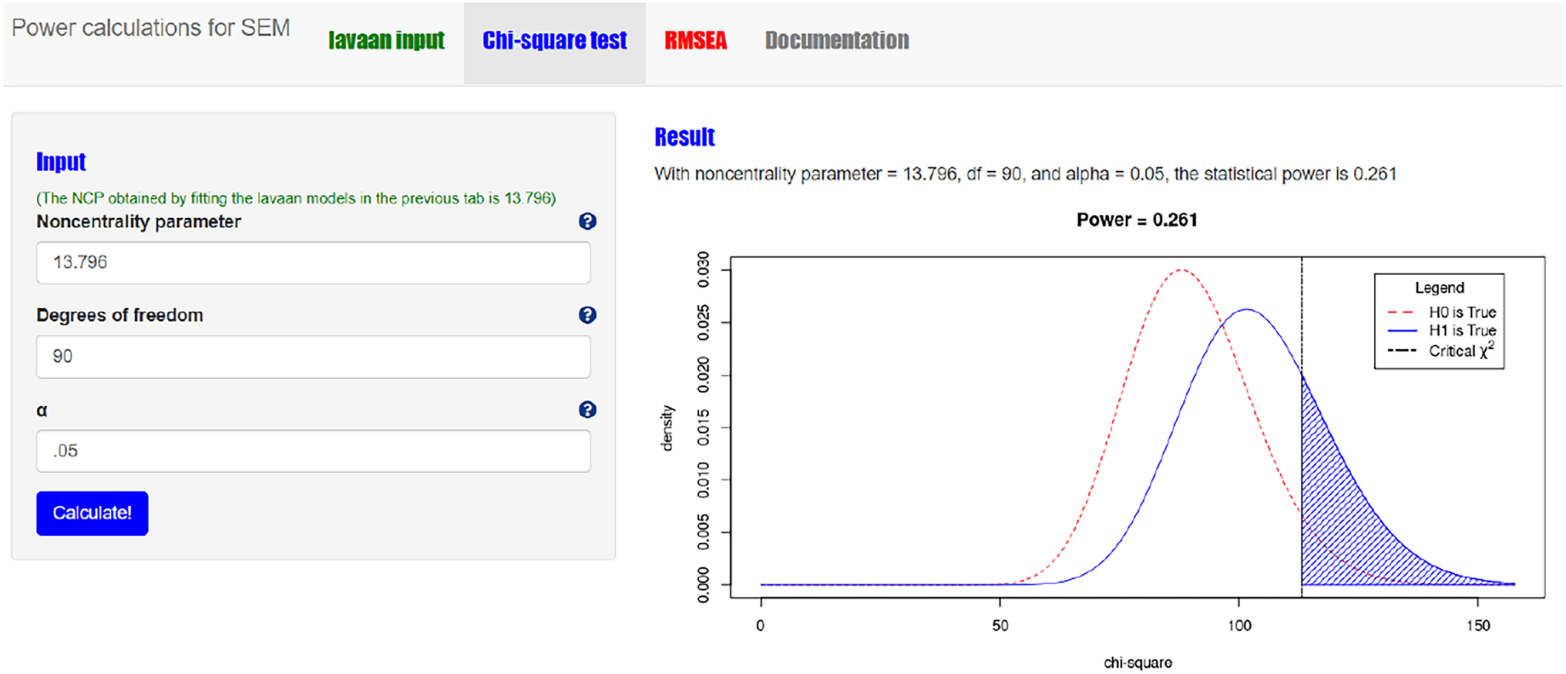
Screenshot of the chi-square based power calculation result for the test on overall model fit of the measurement model with power4SEM

Second, go to the “Chi-square test” page and insert the following values in the box “Input” on the upper left side of the screen: the noncentrality parameter (NCP) value obtained in the first step (i.e., 13.796), the degrees of freedom (Df) of the measurement model (i.e., the number of free statistics minus the number of free model parameters, in our illustrative example this is 152–62 = 90), and the alpha-value (α = 0.05). Click on the blue button “Calculate!”. The result of the power-calculation is now shown both numerically and graphically at the right-hand side of the screen (see Fig. 4). That is, the statistical power to correctly reject our H_0_ model as specified in Fig. 1, when in reality the true model includes two medium-sized cross-loadings, is 0.261. In other words, there is a 26.1% chance of correctly rejecting H_0_. A rather disappointing result considering that one generally wants to achieve a power of 80%.

#### Sample size needed to acquire sufficient power

An additional feature of power4SEM is that it can also be used to calculate the minimum sample size to achieve a desired power of 80%. If we fill in the required values in the box at the bottom left of the “Chi-square test” page, we find that for our illustrative example the minimum sample size needed is 560. In other words, to increase our confidence that the chi-square test of exact fit will reject our model in Fig. 1 when it is misspecified (as defined by two medium-sized cross loadings), we should fit the model to data from at least 560 participants.

The illustrated chi-square based power calculations can thus be a valuable tool in two situations. First, it can be used as a helpful tool for studies in which the sample size is already determined. That is, one can use chi-square based power calculations to provide insight into which model misspecification a study with a given sample size is sensitive to detect (e.g., with 80% power). This is referred to as sensitivity power analysis [25] and is also helpful at the stage of study design as it can show how different sizes of the study sample affect the size of misspecification that one is able to detect with sufficient statistical power. Sensitivity power analyses are not to be confused with post hoc power analyses where one calculates the power to detect an effect with a given size (the effect-size that was found) with a given sample size (the sample size obtained); post hoc power analyses are generally inappropriate [26]. In addition, and preferably, power calculations can be helpful for sample-size planning at the stage of study design (i.e., a priori power calculation) as it will provide information on the required sample size to detect the misspecification of interest with sufficient power. A general drawback of chi-square based power calculations, however, is that it requires explicitly specified models with values for all model parameters.

A more general critique on the use of the chi-square test for overall model fit evaluation is that the null hypothesis of exact fit is invariably false in practical situations, i.e., that the idea that the measurement model fits the data ‘exactly’ is highly implausible. Therefore, there exist a number of alternative model fit indices that intend to give a more descriptive evaluation of how well the model fits the data. The most popular descriptive fit index is the Root Mean Square Error of Approximation (RMSEA; [27]). With this fit index, the null hypothesis of exact fit is replaced by the hypothesis of approximate fit, where it assumed that the specified model will only be an approximation to reality. An RMSEA of zero indicates exact fit, but in evaluating the value of the RMSEA we accept some error of approximation. Browne & Cudeck [28] suggest that an RMSEA below 0.05 indicates “close fit”, an RMSEA between 0.05 and 0.08 is thought to indicate a “reasonable error of approximation”, and that models with an RMSEA above 0.10 have poor fit. An further advantage is that there is also the option to base power calculations for overall model fit evaluation on RMSEA fit index [29].

### RMSEA-based power calculation for overall model fit evaluation

Because the RMSEA value is derived from the chi-square value we can also derive the chi-square distributions under H_0_ and H_1_ from an RMSEA value. That is, in order to calculate statistical power for overall model fit evaluation, we only need to specify the RMSEA-values of H_0_ and H_1_, instead of having to specify all model parameters. So, for example, one can investigate the power to reject close fit (RMSEA value H_0_ = 0.05) when in the population there is not close fit (RMSEA value H_1_ = 0.08). This power calculation is similar to the chi-square-based power calculation in that it provides the power to correctly reject a misspecified measurement model; the difference is that the H_0_ of exact fit is replaced with a H_0_ of close fit. Another advantage of the RMSEA-based power calculation, is that we can also switch the direction of hypothesis testing so that we can calculate the power to reject H_1_ when H_0_ is true. This is an advantage because with SEM we usually believe that H_0_ is true. That is, we believe that the model that we specify under H_0_ is the true model and so we are not directly interested in the power to reject H_0_ when in fact H_1_ is true; but, instead, it would be more informative to know the power to reject H_1_ when H_0_ is true. So, for example, we can investigate the power to reject a model with not-close fit (RMSEA value H_0_ = 0.08) in favor of a model with close fit (RMSEA value H_1_ = 0.05), when there is ‘true’ close fit of the model. More stringently, following MacCallum et al. [29] one could calculate the power to reject a model with ‘not close fit’, using RMSEA H_0_ = 0.05 and RMSEA H_1_ is 0.01. This will give us the probability to correctly reject a model with RMSEA > 0.05 if the population RSMEA is 0.01. Different values may be chosen for H_0_ and H_1_, which will of course impact the calculated power. As a general recommendation, one could use the cut-off values that one uses to base a decision on whether the model does or does not fit well to the data.

#### Step 1: RMSEA-based power calculation with power4SEM

**Fig. 5:**
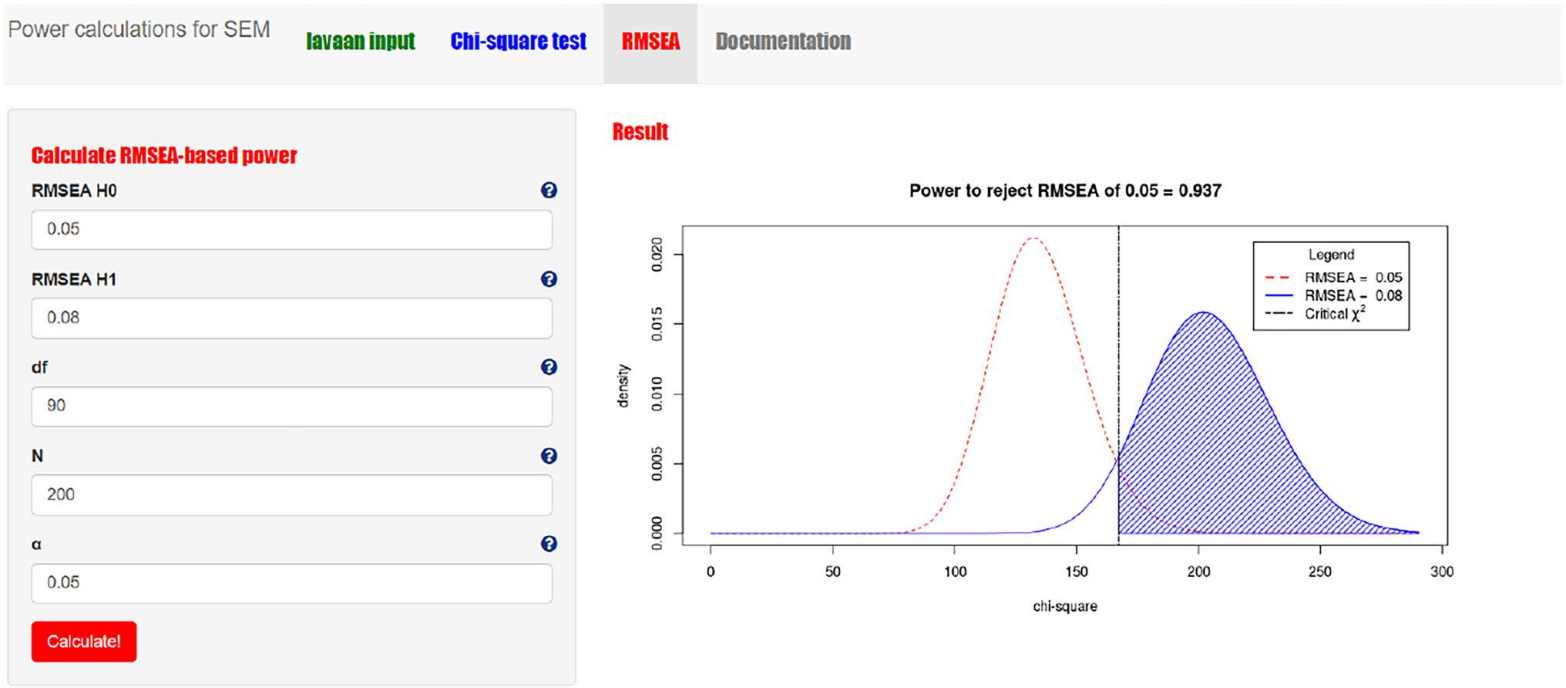
Screenshot of the RMSEA-based power calculation result for the hypothesis of close-fit of the measurement model with power4SEM

RMSEA-based power calculations are also available in the power4SEM app, under the “RMSEA” page. Here, we need to provide the RMSEA-values for H_0_ and H_1_. Suppose we calculate the power to reject close fit (RMSEA = 0.05) of the measurement model in Fig. 1, when there is ‘true’ not-close fit in the population (RMSEA = 0.08). We also provide the intended sample size (N = 200), alpha value (0.05), and number of degrees of freedom of the model of interest (df = 90). If we click on the red button “Calculate!” the result is now shown both numerically and graphically at the right-hand side of the screen (see Fig. 5). When the model in reality shows not-close fit, the power to reject the hypothesis of close-fit is 0.937. If we reverse the RMSEA-values, we will see that the power to reject the hypothesis of not-close fit (RMSEA H_1_ = 0.08) when the model in the populations shows close fit (RMSEA H_0_ = 0.05) is 0.936.

### Step 2: chi-square based power to detect the overall presence of response shift

The second step in the SEM approach for response shift detection entails an omnibus test on the presence of response shift. The presence of response shift is indicated by a change in the pattern of factor loadings (reconceptualization), the value of factor loadings (reprioritization) or the values of intercepts (recalibration^1^) (for more explanation regarding these operationalization of response shift, see [16]). The omnibus test is performed by comparing the so-called ‘no response shift model’, i.e., a model in which all parameters that are associated with response shift are restricted to be equal across time, to the measurement model (in which all these parameters are free to vary across time). The chi-square values of both models can be compared using a chi-square difference test, where a significant *p*-value indicates that H_0_ (no response shift) should be rejected (see also Table 1). In other words, it indicates the overall presence of (any type of) response shift. Statistical power for this chi-square difference test will indicate the chance of correctly rejecting H_0_ (no response shift) when in reality response shift effects are present (see also Table 1). When statistical power is low, there is a high chance that the test will incorrectly indicate that there is no response shift. The difficulty for the power calculation is—similar to Step 1—to define H_1_. Here, H_1_ refers to a model that includes indications of response shift, and one thus has to determine what the ‘overall presence of response shift’ looks like. That is, to determine the exact type, number, and size of possible response shift effects for which H_0_ should be rejected.

#### Specification of H_0_ and H_1_ for the Step 2 chi-square based power calculation

**Fig. 6:**
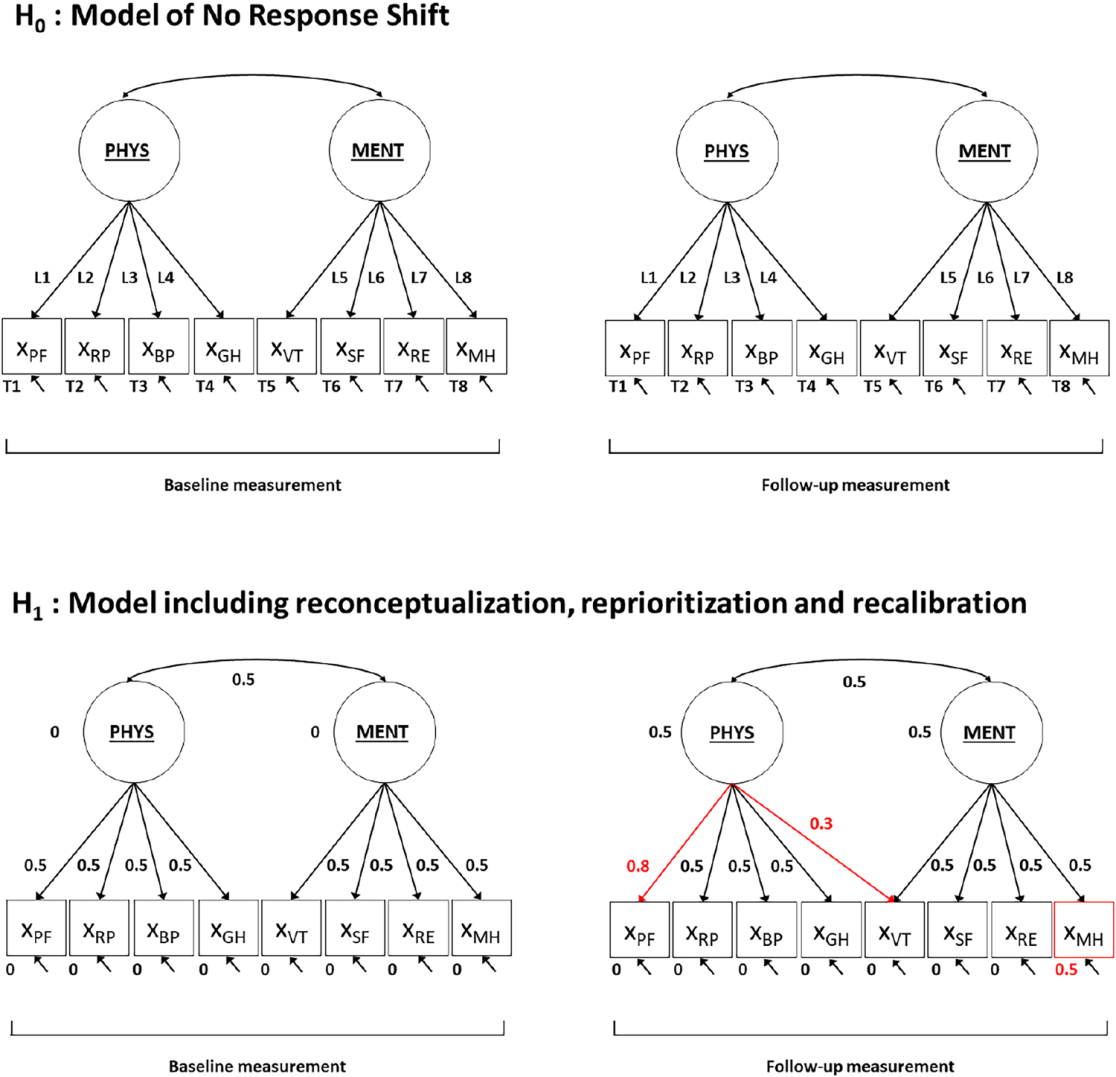
Null-hypothesis (H_0_) model and alternative hypothesis (H_1_) model, including values for the H_1_ model parameters, used in power calculations for the chi-square test applied in step 2 of the SEM approach for response shift detection. *Notes*: The underlying latent variables general physical health (PHYS) and general mental health (MENT) are measured by the observed variables (X), i.e., the subscale scores of the SF-36: physical functioning (PF); role physical (RP); bodily pain (BP); general health (GH); vitality (VT); social functioning (SF); role emotional (RE); mental health (MH). The arrows from the underlying latent variables to the observed variables represent factor loadings, including (for H_1_) parameter values. The arrows between the underlying latent variables represent latent factor correlations at the same occasion, including (for H_1_) parameter values. The single-headed arrows at the bottom of each observed variable represent residual factor variances. The values at the bottom of the observed variables (with H_1_) represent intercept values, and the values next to the underlying latent factors (with H_1_) represent latent factor means. The red parameter values refer to the response shift effects present in H_1_. Note that the longitudinal relations between underlying latent factors and residual factors of the same observed variable are not depicted here for reasons of conciseness (but see Fig. 1)

The H_0_ model that is used in power calculations for the omnibus test on response shift is the ‘no response shift model’ in which all factor loadings and intercepts are restricted to be equal across baseline and follow-up (see Fig. 6 and Appendix II). The number of degrees of freedom for this model are 102 (see Appendix II for more details). The degrees of freedom for the chi-square difference test that is used to test for the overall presence of response shift is thus 102–90 = 12. The H_1_ model is specified the same as the H_0_ model, but includes some response shift effects. That is, the H_1_ model is defined by including differences in the pattern of factor loadings, values of factor loadings and/or intercepts across time. The choice on the type, number, and size of possible response shift effects to include in H_1_ is greatly facilitated when there exist a-priori hypotheses on the potential occurrence of response shift. Based on theory or prior research one may have an idea of what type (i.e., recalibration, reprioritization or reconceptualization), what number, and how large the possible response shift effects may be. For example, previous studies on response shift with the SF-36 indicated the presence of reconceptualization (GH subscale [30]), reprioritization (SF subscale [30], RP subscale [24]) and recalibration response shift (PF subscale [31], RP and BP subscales [30]). When there is no a-priori information available, the specification of a plausible H_1_ is more difficult. As a general recommendation, one could include the minimum number of response shift effects that would be of interest. As the response shift effects refer to targeted parameters, general accepted rules of thumb for the size of the effects can be used to specify small, medium or large effects respectively. The choice of H_1_ model specification in our illustrative example is not based on previous findings of (size of) effects, as the lack of context complicates using substantive considerations in our model specification. Therefore, in our illustrative example H_1_ is specified as a model that includes a total of three response shift effects, i.e., one medium-sized recalibration, reprioritization and reconceptualization effect respectively (see Fig. 6 and Appendix II).

#### Step 2 chi-square based power calculation with power4SEM

**Fig. 7:**
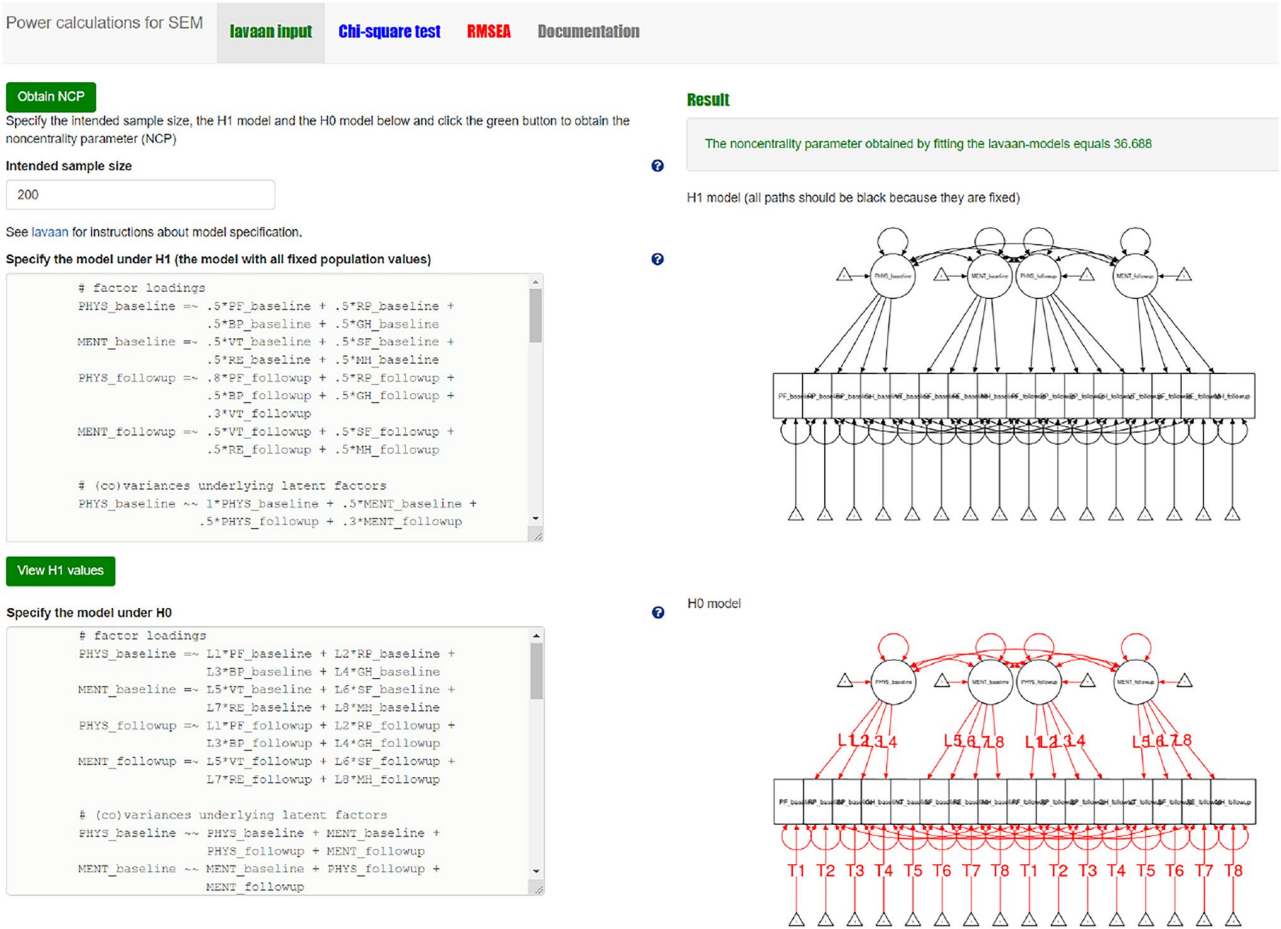
Screenshot of the first step in chi-square based power calculation for the test on overall presence of response shift with power4SEM: inserting the H_0_ and H_1_ model syntax

**Fig. 8:**
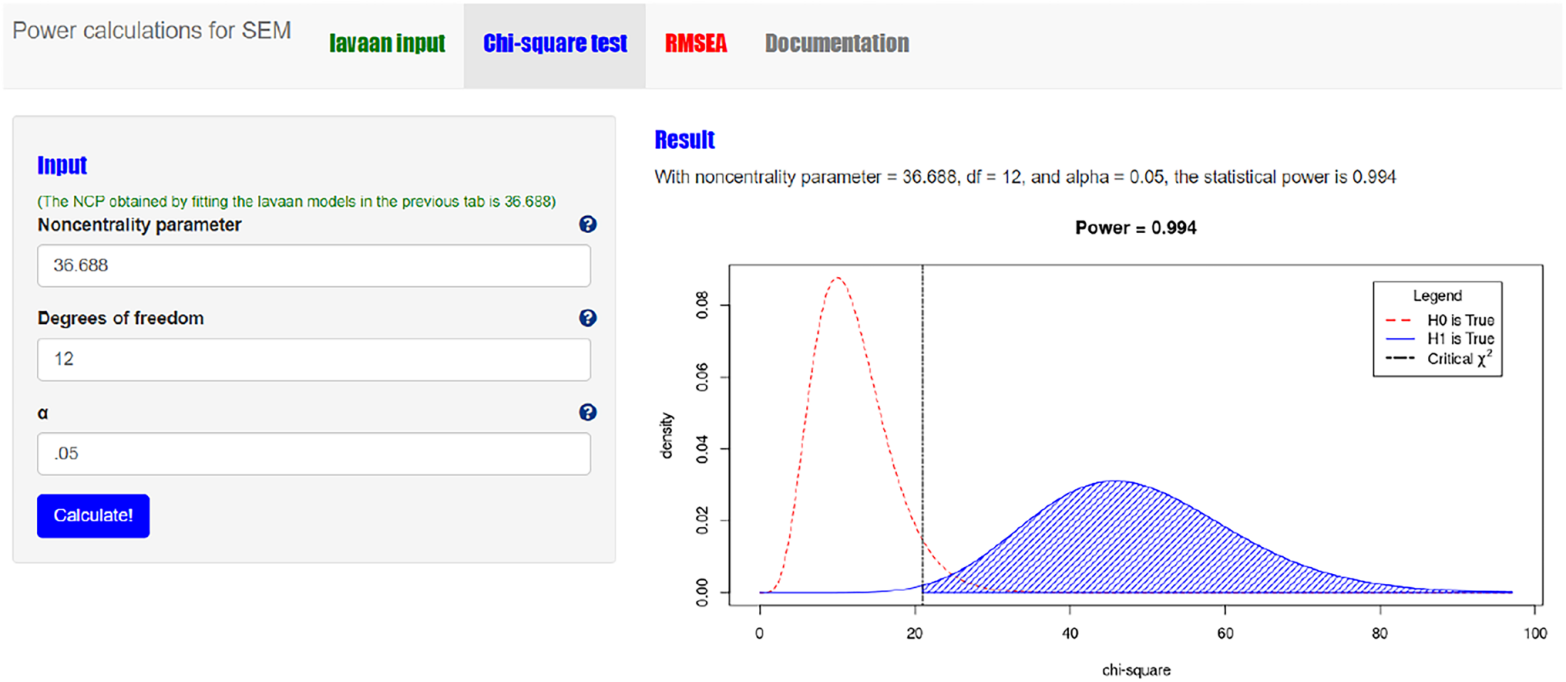
Screenshot of the of the chi-square based power calculation result for the test on overall presence of response shift with power4SEM

When both H_0_ and H_1_ models are specified, and plausible values for all model parameters of H_1_ are provided, we can use power4SEM to calculate the chance to correctly reject H_0_ of no response shift (see also Appendix II). First, the lavaan syntax of the H_0_ and H_1_ models are inserted into the designated input-boxes on the “lavaan input” page (see Fig. 7). The result is obtained by clicking on the green button “Obtain NCP”. Second, on the “Chi-square test” page the obtained NCP-value (36.688), the Df of the chi-square difference test (12), and the appropriate alpha (0.05) are provided as input to obtain the statistical power of the test. The result is shown on the right side of the page (see Fig. 8), where the power to correctly reject H_0_ of no response shift is 0.994. Thus, when there exist three medium-sized response shifts in reality, the omnibus test for response shift is very likely to correctly reject the hypothesis of no response shift.

### Step 3: chi-square based power to detect specific response shift effects

The third step in the SEM approach for response shift detection includes specific tests for response shift effects. That is, the tenability of equality restrictions on model parameters associated with response shift are investigated one by one. Again, the chi-square difference test can be used to test the tenability of the equality restriction. The H_0_ of no response shift now refers to one specific response shift effect (see Table 1). When the *p*-value falls below the alpha-criterion the H_0_ of no response shift specific to the parameter is rejected. Sufficient statistical power is needed to ensure that when the specific response shift effect that is being evaluated exist, that there is a high chance that the chi-square difference test will detect it. If statistical power is low, there is high chance that response shift effects are missed.

#### Specification of H_0_ and H_1_ for the Step 3 chi-square based power calculation

**Fig. 9:**
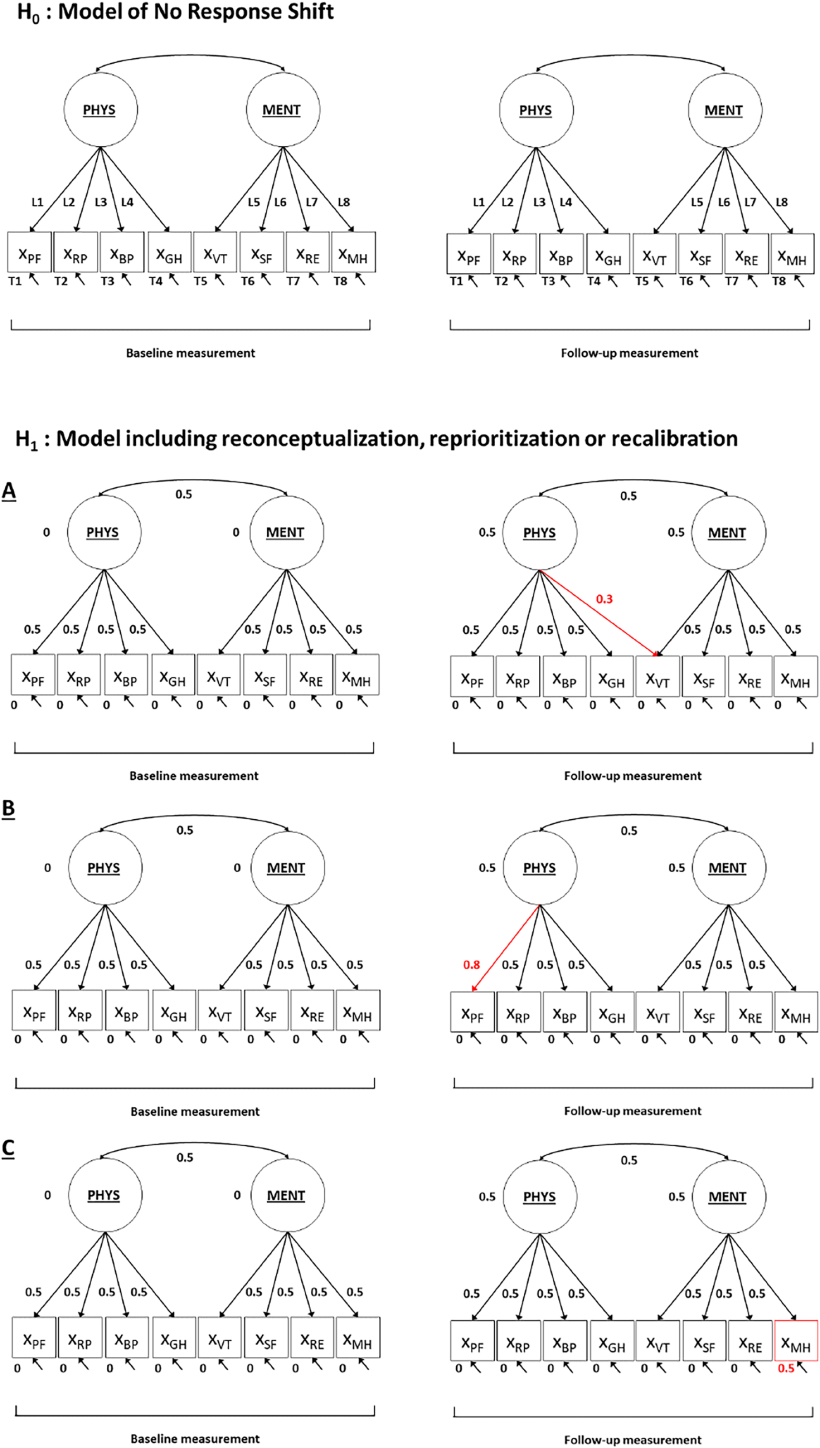
Null-hypothesis (H_0_) model and three alternative hypothesis (H_1_) models (A, B and C), including values for the H_1_ models’ parameters, used in power calculations for the chi-square test applied in step 3 of the SEM approach for response shift detection. *Notes*: The underlying latent variables general physical health (PHYS) and general mental health (MENT) are measured by the observed variables (X), i.e., the subscale scores of the SF-36: physical functioning (PF); role physical (RP); bodily pain (BP); general health (GH); vitality (VT); social functioning (SF); role emotional (RE); mental health (MH). The arrows from the underlying latent variables to the observed variables represent factor loadings, including (for all three H_1_ models) parameter values. The arrows between the underlying latent variables represent latent factor correlations at the same occasion, including (for H_1_ models) parameter values. The single-headed arrows at the bottom of each observed variable represent residual factor variances. The values at the bottom of the observed variables (with H_1_ models) represent intercept values, and the values next to the underlying latent factors (with H_1_ models) represent latent factor means. The red parameter values refer to the response shift effects present in H_1_ models. Note that the longitudinal relations between underlying latent factors and residual factors of the same observed variable are not depicted here for reasons of conciseness (but see Fig. 1)

The H_0_ model that is used in power calculations for tests on specific response shift effects is—again—the ‘no response shift model’ in which all factor loadings and intercepts are restricted to be equal across baseline and follow-up (see Fig. 9 and Appendix III). The difference with the power calculations for the omnibus test for response shift is that the H_1_ model includes only one specific response shift effect. The degrees of freedom for the chi-square difference test that is used to test for the presence of a single response shift is 1 (instead of 12 for the omnibus test of response shift). Using the illustrative example, we specify three different H_1_ models for the detection of one medium-sized recalibration, reprioritization or reconceptualization response shift respectively (see Fig. 8). In this situation there are thus three different power calculations associated with the chi-square test for specific response shift. Here, we elaborate on the power to detect a specific indication of reconceptualization response shift (but see Appendix III for syntaxes of all three power calculations), which is defined as a medium-sized cross-loading of VT at follow-up measurement (H_1_ model A in Fig. 9).

#### Step 3 chi-square based power calculation with power4SEM

**Fig. 10:**
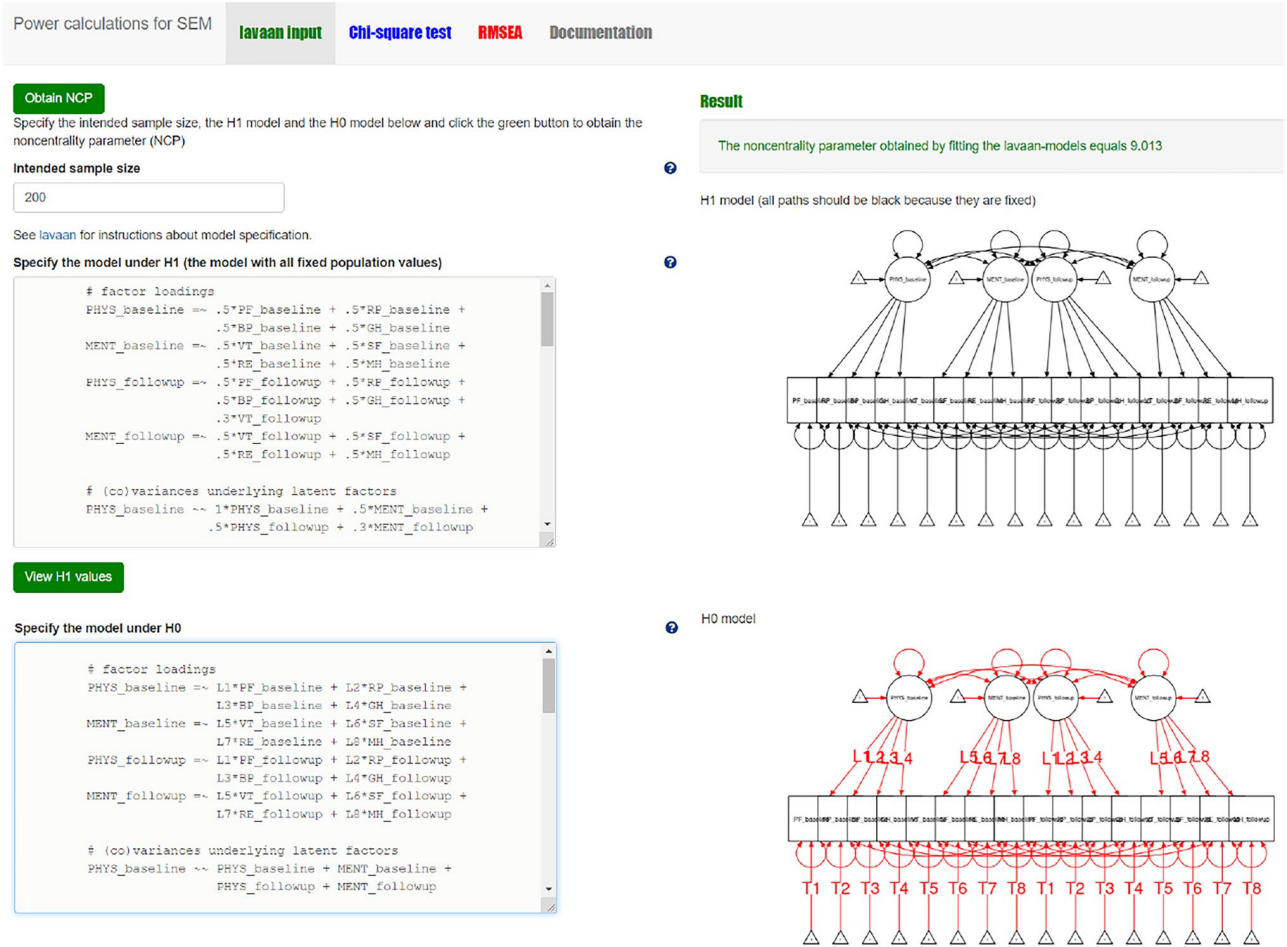
Screenshot of the first step in chi-square based power calculation for test on specific response shift with power4SEM: inserting the H_0_ and H_1_ model syntax

**Fig. 11:**
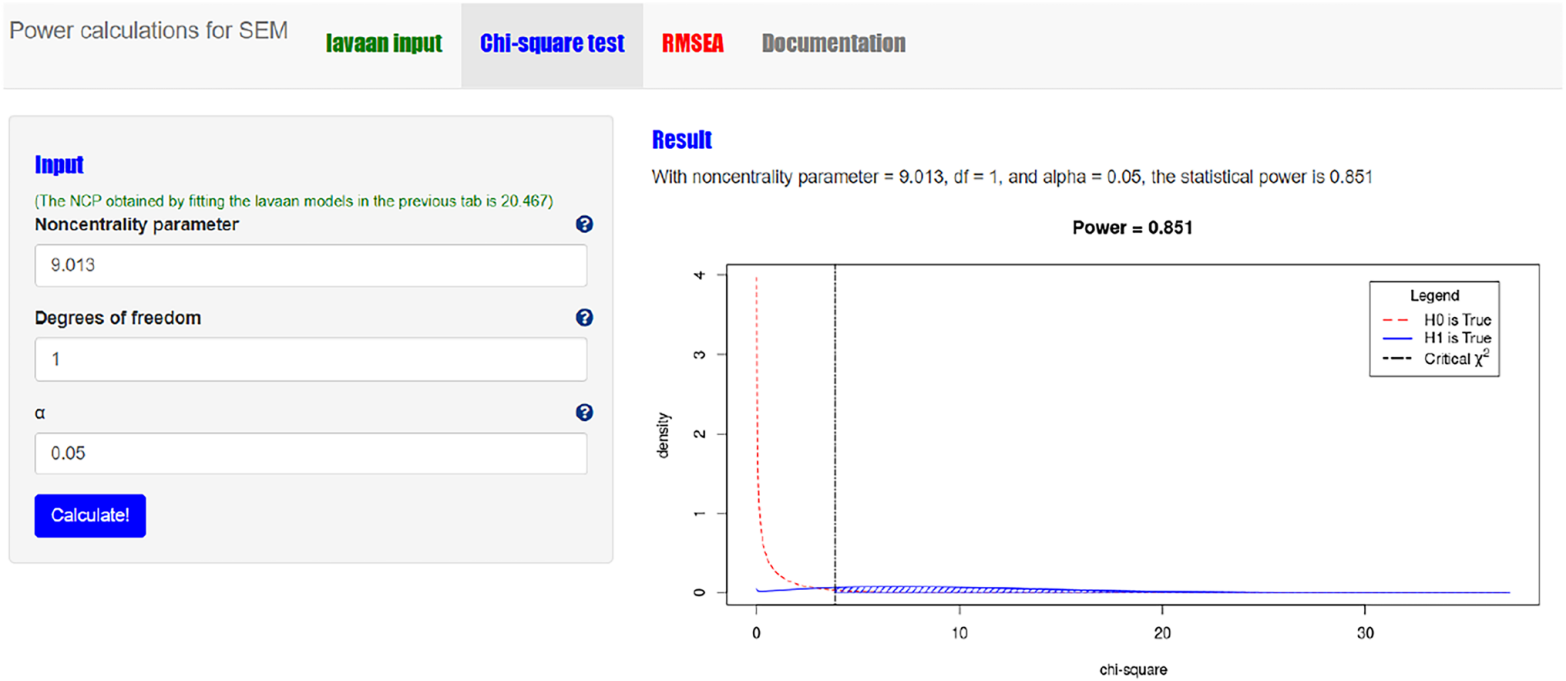
Screenshot of the of the chi-square based power calculation result for the test on specific response shift with power4SEM

We use power4SEM to calculate the chance to correctly reject H_0_ of no response shift, in favor of H_1_ with one indication of a medium-sized reconceptualization response shift (see also Appendix III). The NCP value that is derived by inserting the H_0_ and H_1_ model syntaxes in the “lavaan input” page is 9.013 (see Fig. 10). In combination with Df = 1 and α = 0.05 this results in a power of 0.851 (see Fig. 11). That is, the chance that the H_0_ of no reconceptualization response shift of VT will be correctly rejected (when there is a medium-sized effect present in reality) is 85.1%. This is good news, as the calculated power falls above the desired power of 80%.

Note, that when the omnibus test of response shift is used in the same situation (i.e., when only one reconceptualization response shift is present in reality), the power to detect such an effect is reduced to 45.4% (see Appendix III for details). That is, the power to detect a single response shift effect will be higher for the chi-square test on a specific parameter (i.e., Step 3 of the SEM approach) than it will be for the omnibus chi-square test (i.e., Step 2 of the SEM approach). However, as there are many specific parameters that can be tested for the presence of response shift the increasing number of statistical tests performed on the same data will generally lead to an increased Type I error rate (see Table 1). There is thus a balance to be found between the protection against Type I errors with the omnibus test and the higher power to detect single indications of response shift of the specific test.

## Discussion

The current paper illustrated power calculations for the different steps of the SEM approach to investigate response shift. First, power calculations were illustrated for overall model fit evaluation of the measurement model (i.e., step 1 of the SEM approach). Chi-square based power calculations require the specification of an alternative measurement model that defines the amount of misspecification that one wants to be able to detect (the effect-size value), including all model parameter values. The resulting power can be interpreted as the probability that the hypothesis of exact fit of the measurement model will be rejected, when the alternative measurement model holds in the population. Next to chi-square based power, one can also use RMSEA-based power. One advantage of RMSEA-based power is that it does not require exact model specifications, but instead relies on the RMSEA-values associated with the two measurement models. The resulting power can be interpreted as the probability to reject the hypothesis of close fit of the measurement model, when in reality the measurement model shows not-close fit. Another advantage of RMSEA-based power is that you can flip the hypothesis, so to retrieve the power to reject the hypothesis of not-close fit of the measurement model when in reality the measurement model shows close fit. The latter type of power is most relevant in practice, as the hypothesis is usually that the specified measurement model is the correct model. A drawback, however, is that the amount of misfit in the measurement model as defined by RMSEA-values is hard to interpret. Although chi-square based power calculations are more complicated, it is also more intuitive to think about misfit in terms of specific parameters (e.g., additional factor loadings). Besides practical considerations, it is important to align the type of power calculation with the context and purpose of the study. For example, in a study where a misspecification the pattern of factor loadings is considered to be an important risk, chi-square based power analysis may be most suitable. Or, in the context of competing theoretical models regarding the measurement structure, chi-square based power can be used to calculate the power to reject one theoretical model in favor of the other. RMSEA-based power is most appropriate when specific hypotheses about the measurement structure are absent, and the objective is to find a model that describes the data approximately well. It may informative to use and report the two types of power calculations, with several types of model misspecifications, to provide insight in the sensitivity of the study to detect misspecification.

Chi-square based power calculations were also illustrated for the detection of response shift (i.e., steps 2 and 3 of the SEM procedure). As the hypotheses about the presence of response shift do refer to specific parameters (and not just a general notion of model misfit), it is relatively easy to derive explicitly specified models. Also, as one generally expects that there is some indication of response shift, the resulting power in terms of probability to detect response shift when it is present in the population is directly relevant. However, a difficulty for power calculations and sample size planning for response shift detection with SEM is that it includes different types of power, i.e., power to detect misspecification in the measurement model and power to detect response shift. The different types of power may require very different sample sizes, such that a SEM analysis may be well-powered to detect a model misspecification in the measurement model, but poorly powered to detect response shift, or vice versa. Moreover, although the test on overall presence of response shift (i.e., step 2) and the test for specific indications of response shift tests (i.e., step 3) share the aim to detect possible response shift effects, they do not share the same focus on power to detect effects. That is, the omnibus test may lack power to detect specific indications of response shift, but generally protects against false positives (Type I error). Finding a balance between confidence in the appropriate of the measurement model, desired statistical power to detect response shift, and protection against false positives, is challenging. The different power calculations do provide insight into this balancing act. Another possibility is to consider compromise power analyses, an alternative power analysis in which statistical power and risk of Type I errors are balanced [14, 32, 33].

In general, the factors that affect power in SEM include well-known factors that affect power in any method, like sample size. Other, less well-known factors that influence power in SEM include the distribution of the data, the number and reliability of indicators, the number of latent variables, and the values of all the other parameters in the model. Arguably, the most difficult part of (chi-square based) power calculations is choosing values for all model parameters. That is, specifying the (alternative) measurement model, and to determine the number, type and size of response shift effects to specify. Generally, it is advised that such decisions are based on existing knowledge from prior research. Statistical rules of thumb about the size of effects (i.e., small, medium, large) can also be used to choose appropriate parameter values for effects of interest. Using the illustrative example, it was shown how relevant literature can be used to make a decision on the specification of an alternative measurement model, and inclusion of response shift effects. The parameter values used in the illustrative example were primarily chosen based on statistical rules of thumb of small, medium and large size. As the size of parameter values determine the computed effect-size relevant for the associated statistical power, different parameter values may lead to different conclusions about achieved power or required sample size. Therefore, I would like to note that some alternative recommendations exist for specifying the size of factor loadings. For example, Tabachnick and Fidell [34] argued that based on some general rules of thumb about sample size and alpha level, a factor loading of at least 0.32 should be considered statistically meaningful. Also, one could rely on the size of factor loadings in relation to explained variation. A factor loading of 0.32 would translate to approximately 10 percent variation of the indicator explained by the underlying factor, and the large-sized factor loadings of 0.50 that were used in our illustration translate to 25 percent explained variance. One could argue that for a relevant indicator at least half of the variance should be explained by the underlying factor, and thus the factor loading must exceed 0.70. By relying on statistical rules of thumb for specifying the values of the factor loadings (and other model parameters) we have chosen to use conservative estimates of population values that may have lowered resulting power estimates (i.e., statistical power is higher with stronger measurement structures and/or larger effects). We would thus like to emphasize the importance of relying on previous research to make ecologically valid decisions regarding parameter values when power calculations are used in practice.

Another approach to power calculations is to use a Monte Carlo simulation study (e.g., [15, 35]). In this approach a large number of datasets is generated under the model corresponding to the alternative hypothesis (H_1_), and the null-hypothesis model (H_0_) is fitted to the generated data. Model fit statistics (i.e., chi-square values) and model parameters can be extracted to calculate the proportion of statistically significant results. This results in an empirical estimate of power. It has the advantage that it can take into account possible nonconvergence of models, and is flexible in handling violated assumptions such as non-normally distributed data. However, it is not suited to include RMSEA-based power, is computationally intensive, and conducting simulations generally requires a substantial level of programming experience and statistical expertise.

Concluding, it is important to consider power of intended and performed statistical analyses in the field of response shift research. Recent developments have made power analyses with SEM more feasible and accessible, and the current paper adds to this literature by providing detailed examples. Ideally, any response shift study with SEM should use power calculations when planning sample sizes, or report the power achieved for already performed analyses. Therefore, it is my hope that this paper advances the use of power analyses in applications of SEM for detection of response shift.

### Supplementary Information

Below is the link to the electronic supplementary material.

Supplementary file1 (DOCX 426 KB)

Supplementary file2 (DOCX 381 KB)

Supplementary file3 (DOCX 1146 KB)

## Footnotes

1 This is also known as uniform recalibration, where non-uniform recalibration refers to a change in residual factor variances. However, the detection of non-uniform recalibration is not relevant for the investigation of mean change in the common factor [16] and therefore rarely considered in the literature. Therefore, here we focus on uniform recalibration only, and for reasons of conciseness refer to it as simply ‘recalibration’.
