# Supplementary material for: Power analyses for measurement model misspecification and response shift detection with structural equation modeling
Source: Qual Life Res. 2024 Mar 1;33(5):1241–56. doi: 10.1007/s11136-024-03605-3 (PMC11045588; doi:10.1007/s11136-024-03605-3)
Supplement: Supplementary file 1 — Supplementary file1 (DOCX 426 KB) [file 11136_2024_3605_MOESM1_ESM.docx]

**Appendix I: Chis-square based power calculations for Step 1 of the SEM approach**

**Step 1: Chi-square based power to detect misspecification in the measurement model**

In this step we calculate the statistical power to reject the hypothesis of exact fit of the measurement model (H_0_), when there is no exact fit (misspecification) in the population (H_1_).

**Specification of H_0_**

H_0_ = The measurement model of the SF-36 with two underlying latent factors, as depicted in Figure 1 of the main manuscript.

→ H_0_ includes no values for parameter estimates nor identification restrictions.

### MODEL H0: Measurement model SF36

# factor loadings

PHYS_baseline =~ PF_baseline + RP_baseline + BP_baseline + GH_baseline

MENT_baseline =~ VT_baseline + SF_baseline + RE_baseline + MH_baseline

PHYS_followup =~ PF_followup + RP_followup + BP_followup + GH_followup

MENT_followup =~ VT_followup + SF_followup + RE_followup + MH_followup

# (co)variances underlying latent factors

PHYS_baseline ~~ PHYS_baseline + MENT_baseline + PHYS_followup + MENT_followup

MENT_baseline ~~ MENT_baseline + PHYS_followup + MENT_followup

PHYS_followup ~~ PHYS_followup + MENT_followup

MENT_followup ~~ MENT_followup

# residual factor (co)variances

PF_baseline ~~ PF_baseline + PF_followup

RP_baseline ~~ RP_baseline + RP_followup

BP_baseline ~~ BP_baseline + BP_followup

GH_baseline ~~ GH_baseline + GH_followup

VT_baseline ~~ VT_baseline + VT_followup

SF_baseline ~~ SF_baseline + SF_followup

RE_baseline ~~ RE_baseline + RE_followup

MH_baseline ~~ MH_baseline + MH_followup

PF_followup ~~ PF_followup

RP_followup ~~ RP_followup

BP_followup ~~ BP_followup

GH_followup ~~ GH_followup

VT_followup ~~ VT_followup

SF_followup ~~ SF_followup

RE_followup ~~ RE_followup

MH_followup ~~ MH_followup

# intercept values

PF_baseline ~ 1

RP_baseline ~ 1

BP_baseline ~ 1

GH_baseline ~ 1

VT_baseline ~ 1

SF_baseline ~ 1

RE_baseline ~ 1

MH_baseline ~ 1

PF_followup ~ 1

RP_followup ~ 1

BP_followup ~ 1

GH_followup ~ 1

VT_followup ~ 1

SF_followup ~ 1

RE_followup ~ 1

MH_followup ~ 1

# underlying latent factor means

PHYS_baseline ~ 1

MENT_baseline ~ 1

PHYS_followup ~ 1

MENT_followup ~ 1

*Notes*: The first part of the syntax shows the specification of the underlying latent factors PHYS_baseline, MENT_baseline, PHYS_followup, and MENT_followup; each measured by four subscales of the SF-36 questionnaire. The first part of the syntax thus specifies the number of factor loadings in the model. The second part specifies the variances and covariances between the underlying latent factors, where all relations between underlying latent factors across time are included. The third part specifies the variances and covariances of the residual factors, where only the residual factors of the same indicator are allowed to correlated across time. The final two parts are related to the mean structure, where the intercept values of the observed variables and the means of the underlying latent factors are specified. Note that the mean structure of the factor model is saturated at this stage and therefore does not influence model fit statistics.

**Specification of H_1_**

H_1_ = An alternative measurement model of the SF-36; here defined as H_0_ with two additional cross-loadings of GH and VT.

→ H_1_ can be specified in many different ways, as the difference between H_0_ and H_1_ is an operationalization of misspecification and thus generally does not refer to specific parameters in the model.

→ H_1_ includes values for all model parameters.

**Specification of standardized values for model parameters**

Parameter values are chosen such that they are in standardized metric, this means that:

→ underlying latent factor variances are defined as 1.

→ residual variances are chosen such that the total variance of each observed variable is 1; e.g. when an observed variable has a loading (L) on one underlying latent factor, the residual variance of the observed variable is 1 – L^2^; when an observed variable loads on two underlying latent factors (f1 and f2) it is 1– L1^2^– L2^2^– 2*L1*L2**r*_f1,f2_, where L1 and L2 are the two different loadings and *r*_f1,f2_ is the correlation between f1 and f2.

Values of factor loadings

→ Standardized factor loadings can be interpreted as correlation coefficients, so that according to Cohen’s rules of thumb values of .1, .3 and .5 can be interpreted as small, medium and large respectively.

→ The factor loadings that are also part of H_0_ are specified as being large (.5).

→ The additional cross-loadings of VT and GH are specified as being medium (.3).

→ Other recommendations on the size of factor loadings may be used (see Discussion section main manuscript).

Values of underlying latent factor correlations

→ Correlations between factors at the same occasion are specified as large (.5).

→ Correlations between factors at different occasions are specified as medium (.3).

Values of residual factor correlations

→ Correlations between residual factors are specified as small (.1).

Mean structure

→ Note that the mean structure does not influence model fit statistics at this stage.

→ Intercept values are specified to be zero at both occasions.

→ Underlying latent factor means are zero at the first occasions, and .5 (medium sized change) at the second occasion.

### Model H1: Alternative Measurement Model SF36

# factor loadings

PHYS_baseline =~ .5*PF_baseline + .5*RP_baseline + .5*BP_baseline +

.5*GH_baseline + .3*VT_baseline

MENT_baseline =~ .5*VT_baseline + .5*SF_baseline + .5*RE_baseline +

.5*MH_baseline + .3*GH_baseline

PHYS_followup =~ .5*PF_followup + .5*RP_followup + .5*BP_followup +

.5*GH_followup + .3*VT_followup

MENT_followup =~ .5*VT_followup + .5*SF_followup + .5*RE_followup +

.5*MH_followup + .3*GH_followup

# (co)variances underlying latent factors

PHYS_baseline ~~ 1*PHYS_baseline + .5*MENT_baseline + .5*PHYS_followup +

.3*MENT_followup

MENT_baseline ~~ 1*MENT_baseline + .3*PHYS_followup + .5*MENT_followup

PHYS_followup ~~ 1*PHYS_followup + .5*MENT_followup

MENT_followup ~~ 1*MENT_followup

# residual (co)variances

PF_baseline ~~ .75*PF_baseline + .1*PF_followup

RP_baseline ~~ .75*RP_baseline + .1*RP_followup

BP_baseline ~~ .75*BP_baseline + .1*BP_followup

GH_baseline ~~ .51*GH_baseline + .1*GH_followup

VT_baseline ~~ .51*VT_baseline + .1*VT_followup

SF_baseline ~~ .75*SF_baseline + .1*SF_followup

RE_baseline ~~ .75*RE_baseline + .1*RE_followup

MH_baseline ~~ .75*MH_baseline + .1*MH_followup

PF_followup ~~ .75*PF_followup

RP_followup ~~ .75*RP_followup

BP_followup ~~ .75*BP_followup

GH_followup ~~ .51*GH_followup

VT_followup ~~ .51*VT_followup

SF_followup ~~ .75*SF_followup

RE_followup ~~ .75*RE_followup

MH_followup ~~ .75*MH_followup

# intercept values

PF_baseline ~ 0*1

RP_baseline ~ 0*1

BP_baseline ~ 0*1

GH_baseline ~ 0*1

VT_baseline ~ 0*1

SF_baseline ~ 0*1

RE_baseline ~ 0*1

MH_baseline ~ 0*1

PF_followup ~ 0*1

RP_followup ~ 0*1

BP_followup ~ 0*1

GH_followup ~ 0*1

VT_followup ~ 0*1

SF_followup ~ 0*1

RE_followup ~ 0*1

MH_followup ~ 0*1

# underlying latent factor means

PHYS_baseline ~ 0*1

MENT_baseline ~ 0*1

PHYS_followup ~ 0.5*1

MENT_followup ~ 0.5*1

**Calculation of degrees of freedom for the chi-square test**

To obtain the statistical power of the chi-square test to detect misspecification in the measurement model, we need to calculate the number of degrees of freedom (Df) of the H_0_ model. This number is requested under the “Chi-square test” page of Power4SEM.

→ Degrees of freedom = Free statistics – Free parameter estimates

→ Free statistics in the variance-covariance matrix can be calculated by *v**(*v*+1)/2, where *v* is the number of observed variables in the model. Here *v* = 16 (i.e. 8 subscale scores measured at two occasions), so that the total number of free statistics in the variance-covariance matrix is: 16*17/2 = 136. In addition, the mean structure adds 16 observed means (8 observed variable means at two occasions). Therefore, the total number of free statistics is 136 + 16 = 152.

→ Model parameters: 16 factor loadings + 16 residual variances + 8 residual covariances + 4 underlying latent factor variances + 6 underlying latent factor covariances + 16 intercepts + 4 underlying latent factor means = 70

→ When the H_0_ model is fitted to the data, 8 parameters need to be assigned fixed values for reasons of identification; therefore the total number of free parameter estimates is 70 – 8 = 62

→ Thus, the number of Df of the H_0_ model = 152 - 62 = 90

**Calculate statistical power of the chi-square test for Step 1 with power4SEM**

**1. Use the “lavaan input” page**


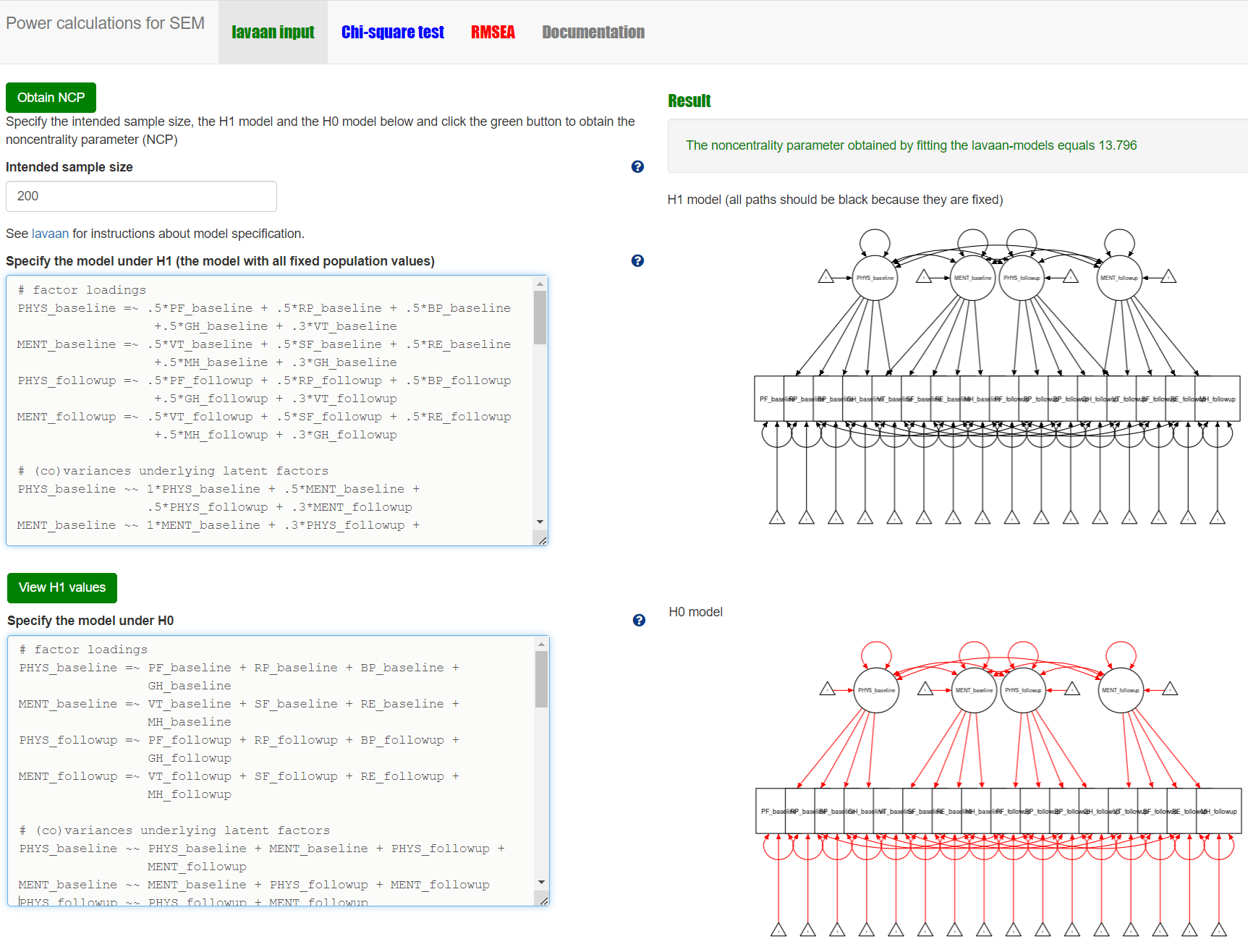


**3. Use N=200 and click “Obtain NCP”**

**2. Insert H_0_ and H_1_ syntax**

**4. Go to the “Chi-square test” page**


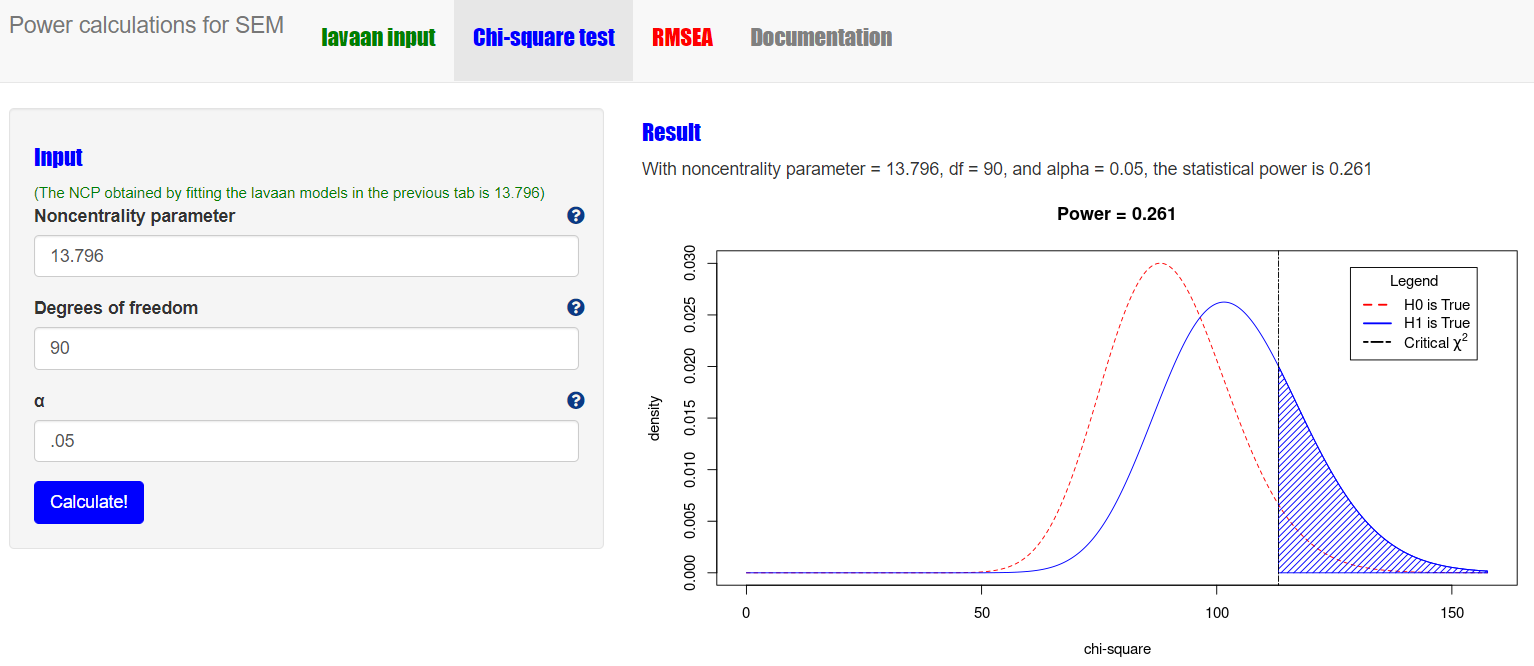


**5. Insert NCP value**

**I**

**6. Insert Df**

**I**

**8. Click “Calculate!”**

**I**

**7. Insert alpha = .05**

**I**
