# Supplementary material for: Power analyses for measurement model misspecification and response shift detection with structural equation modeling
Source: Qual Life Res. 2024 Mar 1;33(5):1241–56. doi: 10.1007/s11136-024-03605-3 (PMC11045588; doi:10.1007/s11136-024-03605-3)
Supplement: Supplementary file 2 — Supplementary file2 (DOCX 381 KB) [file 11136_2024_3605_MOESM2_ESM.docx]

**Appendix II: Chi-square based power calculations for Step 2 of the SEM approach**

**Step 2: Chi-square based power to detect overall response shift**

In this step we calculate the statistical power to reject the hypothesis of no response shift (H_0_), when there is evidence of (overall) response shift in the population (H_1_).

**Specification of H_0_**

H_0_ = The ‘no response shift model’, where all factor loadings and intercepts are restricted to be equal across time

→ The mean structure of the model is now included, as it plays a role in model comparison.

### MODEL H0: Model of no response shift

# factor loadings

PHYS_baseline =~ L1*PF_baseline + L2*RP_baseline + L3*BP_baseline +

L4*GH_baseline

MENT_baseline =~ L5*VT_baseline + L6*SF_baseline + L7*RE_baseline +

L8*MH_baseline

PHYS_followup =~ L1*PF_followup + L2*RP_followup + L3*BP_followup +

L4*GH_followup

MENT_followup =~ L5*VT_followup + L6*SF_followup + L7*RE_followup +

L8*MH_followup

# (co)variances underlying latent factors

PHYS_baseline ~~ PHYS_baseline + MENT_baseline + PHYS_followup + MENT_followup

MENT_baseline ~~ MENT_baseline + PHYS_followup + MENT_followup

PHYS_followup ~~ PHYS_followup + MENT_followup

MENT_followup ~~ MENT_followup

# residual factor (co)variances

PF_baseline ~~ PF_baseline + PF_followup

RP_baseline ~~ RP_baseline + RP_followup

BP_baseline ~~ BP_baseline + BP_followup

GH_baseline ~~ GH_baseline + GH_followup

VT_baseline ~~ VT_baseline + VT_followup

SF_baseline ~~ SF_baseline + SF_followup

RE_baseline ~~ RE_baseline + RE_followup

MH_baseline ~~ MH_baseline + MH_followup

PF_followup ~~ PF_followup

RP_followup ~~ RP_followup

BP_followup ~~ BP_followup

GH_followup ~~ GH_followup

VT_followup ~~ VT_followup

SF_followup ~~ SF_followup

RE_followup ~~ RE_followup

MH_followup ~~ MH_followup

# intercept values

PF_baseline ~ T1*1

RP_baseline ~ T2*1

BP_baseline ~ T3*1

GH_baseline ~ T4*1

VT_baseline ~ T5*1

SF_baseline ~ T6*1

RE_baseline ~ T7*1

MH_baseline ~ T8*1

PF_followup ~ T1*1

RP_followup ~ T2*1

BP_followup ~ T3*1

GH_followup ~ T4*1

VT_followup ~ T5*1

SF_followup ~ T6*1

RE_followup ~ T7*1

MH_followup ~ T8*1

# underlying latent factor means

PHYS_baseline ~ 1

MENT_baseline ~ 1

PHYS_followup ~ 1

MENT_followup ~ 1

*Notes*: The first part of the syntax shows the specification of the underlying latent factors PHYS_baseline, MENT_baseline, PHYS_followup, and MENT_followup; each measured by four subscales of the SF-36 questionnaire. Labels are used to ensure that the factor loadings of the same variable are restricted to be equal across time, e.g. L1 is used to ensure that the factor loading of PF is the same value at both baseline and follow-up occasion. The variances and covariances between the underlying latent factors, and variances and covariances of the residual factors are specified in the same way as for the Step 1 power-calculations (see Appendix I). The mean structure is now included as well, where the intercept values of the same observed variable is restricted to be equal across occasions by using the same labels (T1 through T8). Finally, the syntax includes the specification of the means of the underlying latent factors.

**Specification of H_1_**

H_1_ = A model that includes indications of response shift; here, we define H_1_ as a model that includes one reconceptualization, one reprioritization and one recalibration effect.

→ H_1_ can be specified in many different ways, i.e. one needs to decide on the number, the type, and the size of possible response shift effects to include.

→ H_1_ includes values for all model parameters (see Appendix I for more details on choosing values for model parameters)

**Specification of standardized values for model parameters**

Parameter values are chosen such that they are in standardized metric (see description of Step 1 in Appendix I). This facilitates interpretation, as not only the associations across time between underlying latent factors and residual factors but also the standardized factor loadings can interpreted as correlation coefficients where values of .1, .3, and .5 are indicative of small, medium, and large effects respectively (Cohen, 1988). In addition, with regards to the specification of the mean structure, differences in the intercept values and the means of the underlying latent factors can be interpreted as Cohen’s *d* effect size (Cohen, 1988), where values of .2, .5 and .8 indicate small, medium and large effects respectively.

Values of factor loadings

→ The factor loadings that are also part of H_0_ are specified as being large (.5)

→ The reconceptualization effect is defined as a medium-sized (.3) cross-loading at follow-up occasion of VT

→ The reprioritization effect is defined as a medium-sized change (.3, from .5 to .8) in the value of the factor loading of PF

Values of underlying latent factor correlations

→ Correlations between factors at the same occasion are specified as large (.5)

→ Correlations between factors at different occasions are specified as medium (.3)

Values of residual factor correlations

→ Correlations between residual factors are specified as small (.1)

Values of the intercepts

→ Intercept values are specified to be zero at both occasions

→ The recalibration effect is specified as a medium-sized change (.5) in intercept value of MH

Values of the underlying latent factor means

→ Underlying latent factor means are zero at the first occasion, and .5 (medium sized change) at the second occasion.

### Model H1: Model including reconceptualization, reprioritization and recalibration

# factor loadings

PHYS_baseline =~ .5*PF_baseline + .5*RP_baseline + .5*BP_baseline +

.5*GH_baseline

MENT_baseline =~ .5*VT_baseline + .5*SF_baseline + .5*RE_baseline +

.5*MH_baseline

PHYS_followup =~ .8*PF_followup + .5*RP_followup + .5*BP_followup +

.5*GH_followup + .3*VT_followup

MENT_followup =~ .5*VT_followup + .5*SF_followup + .5*RE_followup +

.5*MH_followup

# (co)variances underlying latent factors

PHYS_baseline ~~ 1*PHYS_baseline + .5*MENT_baseline + .5*PHYS_followup +

.3*MENT_followup

MENT_baseline ~~ 1*MENT_baseline + .3*PHYS_followup + .5*MENT_followup

PHYS_followup ~~ 1*PHYS_followup + .5*MENT_followup

MENT_followup ~~ 1*MENT_followup

# residual (co)variances

PF_baseline ~~ .75*PF_baseline + .1*PF_followup

RP_baseline ~~ .75*RP_baseline + .1*RP_followup

BP_baseline ~~ .75*BP_baseline + .1*BP_followup

GH_baseline ~~ .75*GH_baseline + .1*GH_followup

VT_baseline ~~ .75*VT_baseline + .1*VT_followup

SF_baseline ~~ .75*SF_baseline + .1*SF_followup

RE_baseline ~~ .75*RE_baseline + .1*RE_followup

MH_baseline ~~ .75*MH_baseline + .1*MH_followup

PF_followup ~~ .36*PF_followup

RP_followup ~~ .75*RP_followup

BP_followup ~~ .75*BP_followup

GH_followup ~~ .75*GH_followup

VT_followup ~~ .51*VT_followup

SF_followup ~~ .75*SF_followup

RE_followup ~~ .75*RE_followup

MH_followup ~~ .75*MH_followup

# intercept values

PF_baseline ~ 0*1

RP_baseline ~ 0*1

BP_baseline ~ 0*1

GH_baseline ~ 0*1

VT_baseline ~ 0*1

SF_baseline ~ 0*1

RE_baseline ~ 0*1

MH_baseline ~ 0*1

PF_followup ~ 0*1

RP_followup ~ 0*1

BP_followup ~ 0*1

GH_followup ~ 0*1

VT_followup ~ 0*1

SF_followup ~ 0*1

RE_followup ~ 0*1

MH_followup ~ 0.5*1

# underlying latent factor means

PHYS_baseline ~ 0*1

MENT_baseline ~ 0*1

PHYS_followup ~ 0.5*1

MENT_followup ~ 0.5*1

**Calculate statistical power of the chi-square difference test for Step 2 with power4SEM**

**1. Use the “lavaan input” page**


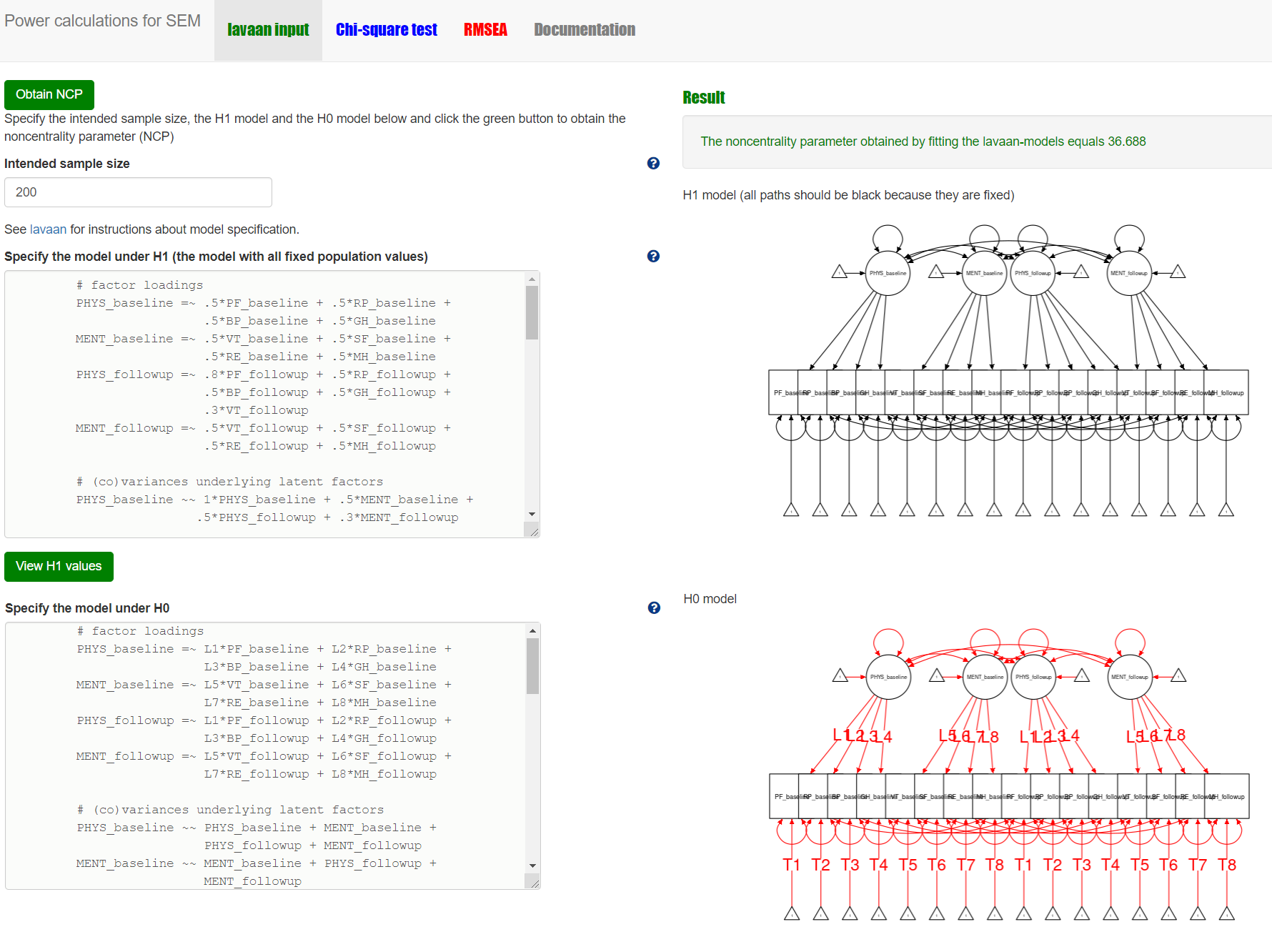


**3. Use N=200 and click “Obtain NCP”**

**2. Insert H_0_ and H_1_ syntax**

**4. Go to the “Chi-square test” page**


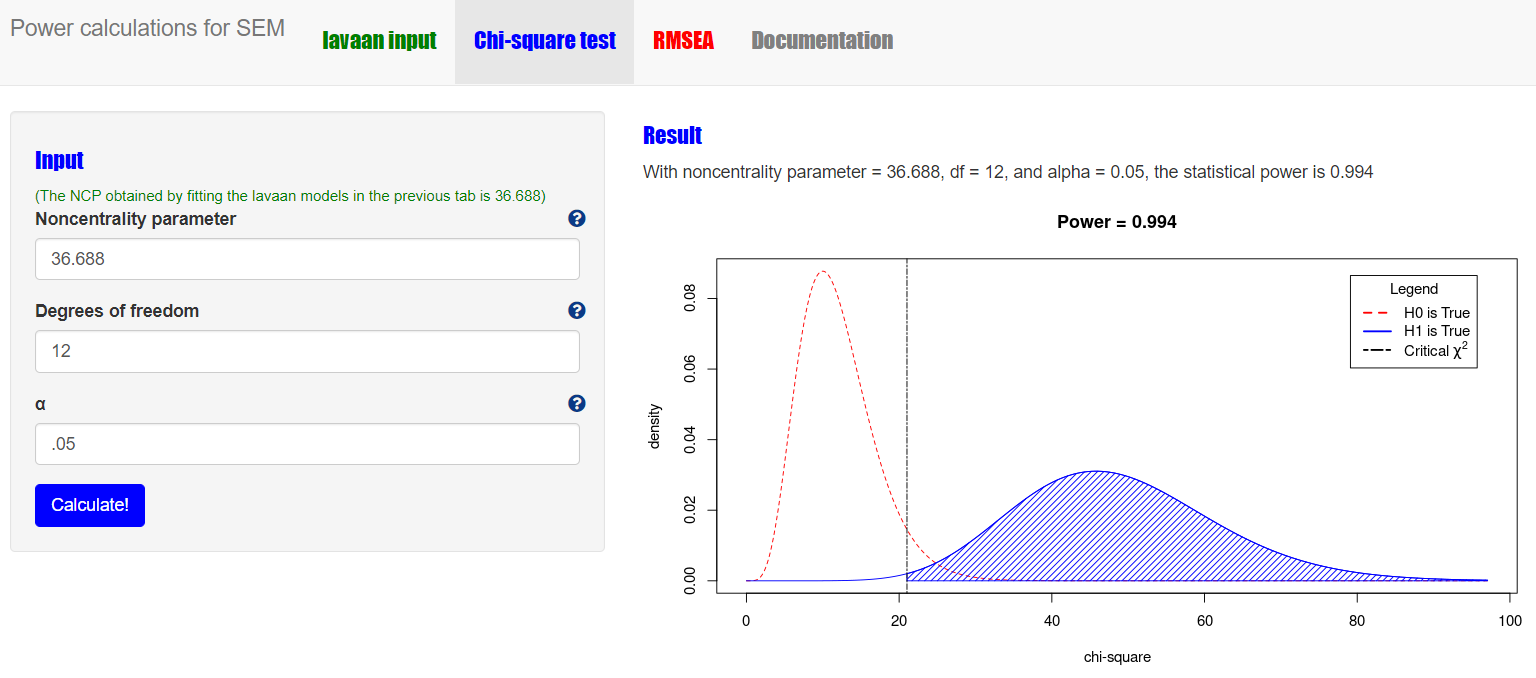


**5. Insert NCP value**

**I**

**6. Insert Df (see below)**

**I**

**8. Click “Calculate!”**

**I**

**7. Insert alpha = .05**

**I**

**Calculation of degrees of freedom for the chi-square difference test**

To obtain the statistical power of the chi-square difference test to detect overall presence of response shift, we need to calculate the number of degrees of freedom (Df) associated with the chi-square difference test. This number is the difference in freely estimated model parameters between the no response shift model and the measurement model. One can derive this number through calculation of the freely estimated model parameters of each model, or alternatively by comparing the number of Df of both models.

→ Freely estimated model parameters measurement model (including mean structure): 16 factor loadings + 16 residual variances + 8 residual covariances + 4 underlying latent factor variances + 6 underlying latent factor covariances + 4 underlying latent factor means + 16 intercepts – 8 identification restrictions = 62

→ Freely estimated model parameters no response shift model: 8 factor loadings + 16 residual variances + 8 residual covariances + 4 underlying latent factor variances + 6 underlying latent factor covariances + 4 underlying latent factor means + 8 intercept values – 4 identification restrictions = 50

→ Thus, the number of Df associated with the chi-square difference test = 62 - 50 = 12

→ Alternatively, the number of Df of the measurement model was 90. The number of Df of the no response shift model is (16*17/2 + 16) – 50 = 102. Thus, the difference between the Df’s of both models equals the difference in the number of freely estimated parameters.

→ Note that in the no response shift model there are less identification restrictions needed due to the equality restrictions on the factor loadings and intercepts across time (see Oort, 2005).
