# Supplementary material for: Power analyses for measurement model misspecification and response shift detection with structural equation modeling
Source: Qual Life Res. 2024 Mar 1;33(5):1241–56. doi: 10.1007/s11136-024-03605-3 (PMC11045588; doi:10.1007/s11136-024-03605-3)
Supplement: Supplementary file 3 — Supplementary file3 (DOCX 1146 KB) [file 11136_2024_3605_MOESM3_ESM.docx]

**Appendix III: Chi-square based power calculations for Step 3 of the SEM approach**

**Step 3: Chi-square based power to detect specific response shift**

In this step, we calculate the statistical power to reject the hypothesis of no response shift in a specific parameter (H_0_), when there is evidence of response shift in this parameter in the population (H_1_).

**Specification of H_0_**

H_0_ = The ‘no response shift model’, where all factor loadings and intercepts are restricted to be equal across time

##

### MODEL H0: Model of no response shift

# factor loadings

PHYS_baseline =~ L1*PF_baseline + L2*RP_baseline + L3*BP_baseline +

L4*GH_baseline

MENT_baseline =~ L5*VT_baseline + L6*SF_baseline + L7*RE_baseline +

L8*MH_baseline

PHYS_followup =~ L1*PF_followup + L2*RP_followup + L3*BP_followup +

L4*GH_followup

MENT_followup =~ L5*VT_followup + L6*SF_followup + L7*RE_followup +

L8*MH_followup

# (co)variances underlying latent factors

PHYS_baseline ~~ PHYS_baseline + MENT_baseline + PHYS_followup + MENT_followup

MENT_baseline ~~ MENT_baseline + PHYS_followup + MENT_followup

PHYS_followup ~~ PHYS_followup + MENT_followup

MENT_followup ~~ MENT_followup

# residual factor (co)variances

PF_baseline ~~ PF_baseline + PF_followup

RP_baseline ~~ RP_baseline + RP_followup

BP_baseline ~~ BP_baseline + BP_followup

GH_baseline ~~ GH_baseline + GH_followup

VT_baseline ~~ VT_baseline + VT_followup

SF_baseline ~~ SF_baseline + SF_followup

RE_baseline ~~ RE_baseline + RE_followup

MH_baseline ~~ MH_baseline + MH_followup

PF_followup ~~ PF_followup

RP_followup ~~ RP_followup

BP_followup ~~ BP_followup

GH_followup ~~ GH_followup

VT_followup ~~ VT_followup

SF_followup ~~ SF_followup

RE_followup ~~ RE_followup

MH_followup ~~ MH_followup

# intercept values

PF_baseline ~ T1*1

RP_baseline ~ T2*1

BP_baseline ~ T3*1

GH_baseline ~ T4*1

VT_baseline ~ T5*1

SF_baseline ~ T6*1

RE_baseline ~ T7*1

MH_baseline ~ T8*1

PF_followup ~ T1*1

RP_followup ~ T2*1

BP_followup ~ T3*1

GH_followup ~ T4*1

VT_followup ~ T5*1

SF_followup ~ T6*1

RE_followup ~ T7*1

MH_followup ~ T8*1

# underlying latent factor means

PHYS_baseline ~ 1

MENT_baseline ~ 1

PHYS_followup ~ 1

MENT_followup ~ 1

*Notes*: The first part of the syntax shows the specification of the underlying latent factors PHYS_baseline, MENT_baseline, PHYS_followup, and MENT_followup; each measured by four subscales of the SF-36 questionnaire. Labels are used to ensure that the factor loadings of the same variable are restricted to be equal across time, e.g. L1 is used to ensure that the factor loading of PF is the same value at both baseline and follow-up occasion. The variances and covariances between the underlying latent factors, and variances and covariances of the residual factors are specified in the same way as for the Step 1 power-calculations. The mean structure is now included as well, where the intercept values of the same observed variable is restricted to be equal across occasions by using the same labels (i.e. T1 through T8). Finally, the syntax includes the specification of the means of the underlying latent factors.

**Specification of H_1_**

H_1_ = A model that includes one indication of response shift; here, we define three different H_1_ that include one reconceptualization, reprioritization or recalibration effect respectively.

→ H_1_ thus refers to a model that includes a specific response shift effect of interest

**Specification of standardized values for model parameters**

Parameter values are chosen such that they are in standardized metric (see Appendices I & II for the descriptions of Steps 1 and 2 and for details on the model parameter values).

Specification of response shift effects

→ The reconceptualization effect is defined as a medium-sized (.3) cross-loading at follow-up occasion of VT

→ The reprioritization effect is defined as a medium-sized change (.3) in the value of the factor loading of PF

→ The recalibration effect is specified as a medium-sized change (.5) in intercept value of MH

**Model 3A: H_1_ that includes one medium-sized reconceptualization effect**

### Model H1.A: Model including one reconceptualization effect

# factor loadings

PHYS_baseline =~ .5*PF_baseline + .5*RP_baseline + .5*BP_baseline +

.5*GH_baseline

MENT_baseline =~ .5*VT_baseline + .5*SF_baseline + .5*RE_baseline +

.5*MH_baseline

PHYS_followup =~ .5*PF_followup + .5*RP_followup + .5*BP_followup +

.5*GH_followup + .3*VT_followup

MENT_followup =~ .5*VT_followup + .5*SF_followup + .5*RE_followup +

.5*MH_followup

# (co)variances underlying latent factors

PHYS_baseline ~~ 1*PHYS_baseline + .5*MENT_baseline + .5*PHYS_followup +

.3*MENT_followup

MENT_baseline ~~ 1*MENT_baseline + .3*PHYS_followup + .5*MENT_followup

PHYS_followup ~~ 1*PHYS_followup + .5*MENT_followup

MENT_followup ~~ 1*MENT_followup

# residual (co)variances

PF_baseline ~~ .75*PF_baseline + .1*PF_followup

RP_baseline ~~ .75*RP_baseline + .1*RP_followup

BP_baseline ~~ .75*BP_baseline + .1*BP_followup

GH_baseline ~~ .75*GH_baseline + .1*GH_followup

VT_baseline ~~ .75*VT_baseline + .1*VT_followup

SF_baseline ~~ .75*SF_baseline + .1*SF_followup

RE_baseline ~~ .75*RE_baseline + .1*RE_followup

MH_baseline ~~ .75*MH_baseline + .1*MH_followup

PF_followup ~~ .75*PF_followup

RP_followup ~~ .75*RP_followup

BP_followup ~~ .75*BP_followup

GH_followup ~~ .75*GH_followup

VT_followup ~~ .51*VT_followup

SF_followup ~~ .75*SF_followup

RE_followup ~~ .75*RE_followup

MH_followup ~~ .75*MH_followup

# intercept values

PF_baseline ~ 0*1

RP_baseline ~ 0*1

BP_baseline ~ 0*1

GH_baseline ~ 0*1

VT_baseline ~ 0*1

SF_baseline ~ 0*1

RE_baseline ~ 0*1

MH_baseline ~ 0*1

PF_followup ~ 0*1

RP_followup ~ 0*1

BP_followup ~ 0*1

GH_followup ~ 0*1

VT_followup ~ 0*1

SF_followup ~ 0*1

RE_followup ~ 0*1

MH_followup ~ 0*1

# underlying latent factor means

PHYS_baseline ~ 0*1

MENT_baseline ~ 0*1

PHYS_followup ~ 0.5*1

MENT_followup ~ 0.5*1

**Calculate statistical power of the chi-square difference test for Step 3 with power4SEM**

**1. Use the “lavaan input” page**


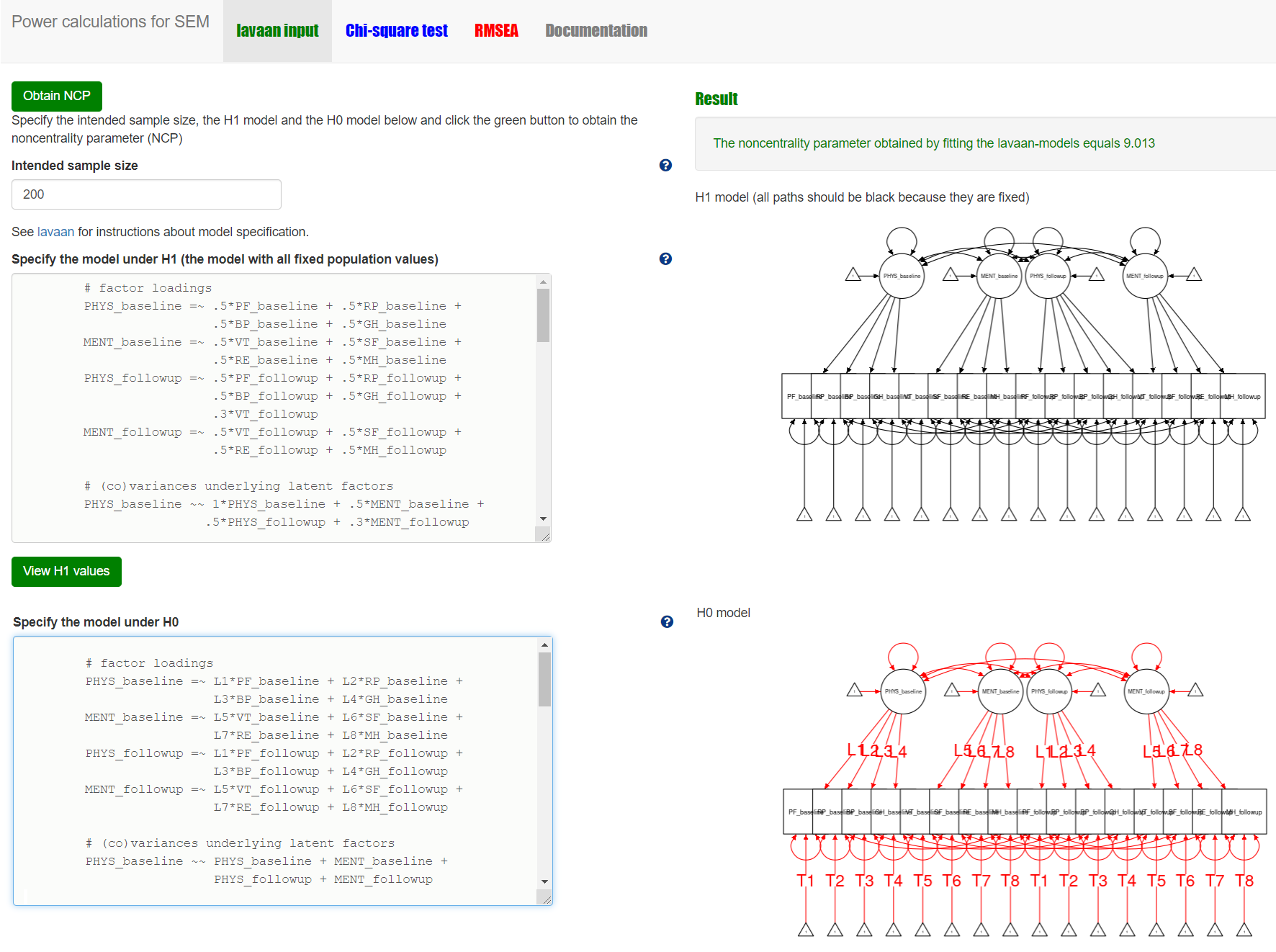


**3. Use N=200 and click “Obtain NCP”**

**2. Insert H_0_ and H_1_ syntax**

**4. Go to the “Chi-square test” page**


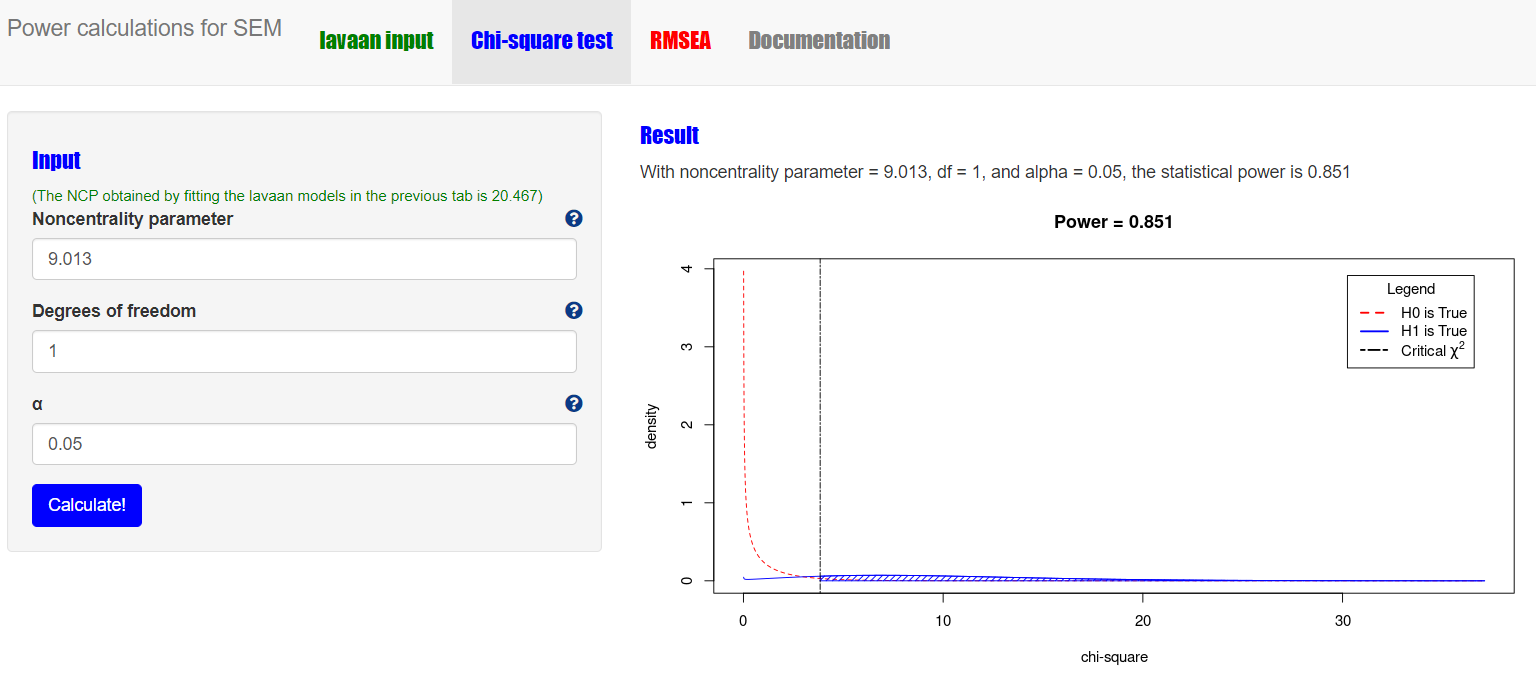


**5. Insert NCP value**

**I**

**6. Insert Df = 1**

**I**

**8. Click “Calculate!”**

**I**

**7. Insert alpha = .05**

**I**

**The power of the chi-square difference test for overall response shift in this example**

To illustrate that the chi-square test for specific response shift has more power to detect response shift as compared to the omnibus test that is used in step 2 of the SEM procedure, here we also include the power-calculation for the omnibus test in the situation where there is only one medium-sized reconceptualization effect in reality (H_1_).


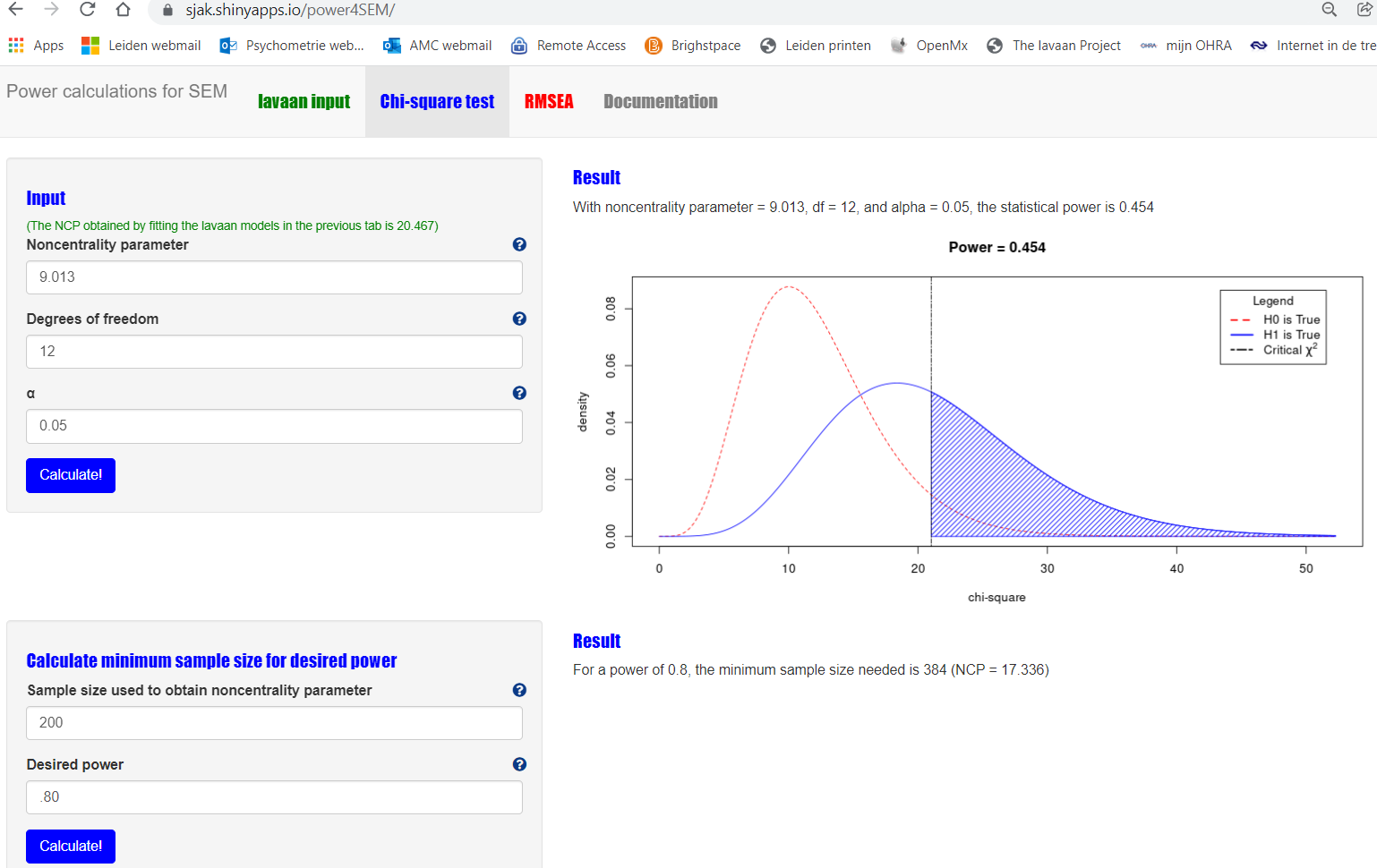


The H_0_ and H_1_ models are the same as are used in the example of the chi-square difference test for specific response shift. The difference is that the omnibus test has Df=12 (as compared to Df=1). This results in an achieved power of .454. Therefore, if there is only one medium-sized reconceptualization effect there will be a 45.4% chance to correctly reject the no response shift model with the omnibus test (instead of 85.1% for the individual test). Note that due to the fact that there are 16 individual response shift effects that can be tested in Step 3, the higher power of the test for specific response shift effects come at a cost of increased type I error rate. If one would adjust the alpha-criterion with, for example, a bonferroni correction than this would of course again decrease the type I error rate (but also the statistical power).

**Model 3B: H_1_ that includes one medium-sized reprioritization effect**

### Model H1.B: Model including one reprioritization effect

# factor loadings

PHYS_baseline =~ .5*PF_baseline + .5*RP_baseline + .5*BP_baseline +

.5*GH_baseline

MENT_baseline =~ .5*VT_baseline + .5*SF_baseline + .5*RE_baseline +

.5*MH_baseline

PHYS_followup =~ .8*PF_followup + .5*RP_followup + .5*BP_followup +

.5*GH_followup

MENT_followup =~ .5*VT_followup + .5*SF_followup + .5*RE_followup +

.5*MH_followup

# (co)variances underlying latent factors

PHYS_baseline ~~ 1*PHYS_baseline + .5*MENT_baseline + .5*PHYS_followup +

.3*MENT_followup

MENT_baseline ~~ 1*MENT_baseline + .3*PHYS_followup + .5*MENT_followup

PHYS_followup ~~ 1*PHYS_followup + .5*MENT_followup

MENT_followup ~~ 1*MENT_followup

# residual (co)variances

PF_baseline ~~ .75*PF_baseline + .1*PF_followup

RP_baseline ~~ .75*RP_baseline + .1*RP_followup

BP_baseline ~~ .75*BP_baseline + .1*BP_followup

GH_baseline ~~ .75*GH_baseline + .1*GH_followup

VT_baseline ~~ .75*VT_baseline + .1*VT_followup

SF_baseline ~~ .75*SF_baseline + .1*SF_followup

RE_baseline ~~ .75*RE_baseline + .1*RE_followup

MH_baseline ~~ .75*MH_baseline + .1*MH_followup

PF_followup ~~ .36*PF_followup

RP_followup ~~ .75*RP_followup

BP_followup ~~ .75*BP_followup

GH_followup ~~ .75*GH_followup

VT_followup ~~ .75*VT_followup

SF_followup ~~ .75*SF_followup

RE_followup ~~ .75*RE_followup

MH_followup ~~ .75*MH_followup

# intercept values

PF_baseline ~ 0*1

RP_baseline ~ 0*1

BP_baseline ~ 0*1

GH_baseline ~ 0*1

VT_baseline ~ 0*1

SF_baseline ~ 0*1

RE_baseline ~ 0*1

MH_baseline ~ 0*1

PF_followup ~ 0*1

RP_followup ~ 0*1

BP_followup ~ 0*1

GH_followup ~ 0*1

VT_followup ~ 0*1

SF_followup ~ 0*1

RE_followup ~ 0*1

MH_followup ~ 0*1

# underlying latent factor means

PHYS_baseline ~ 0*1

MENT_baseline ~ 0*1

PHYS_followup ~ 0.5*1

MENT_followup ~ 0.5*1

**Calculate statistical power of the chi-square difference test for Step 3 with power4SEM**

**1. Use the “lavaan input” page**


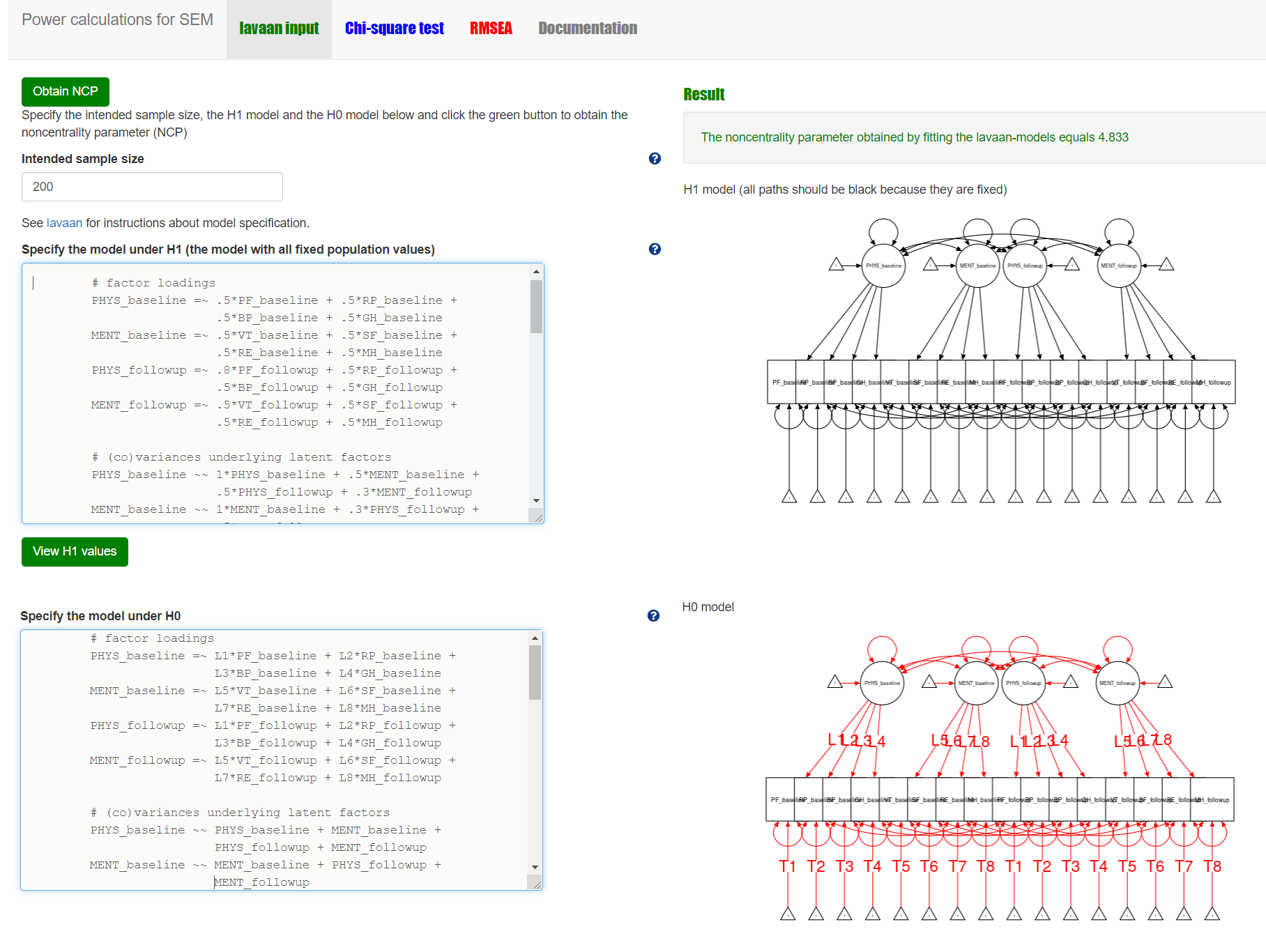


**3. Use N=200 and click “Obtain NCP”**

**2. Insert H_0_ and H_1_ syntax**

**4. Go to the “Chi-square test” page**


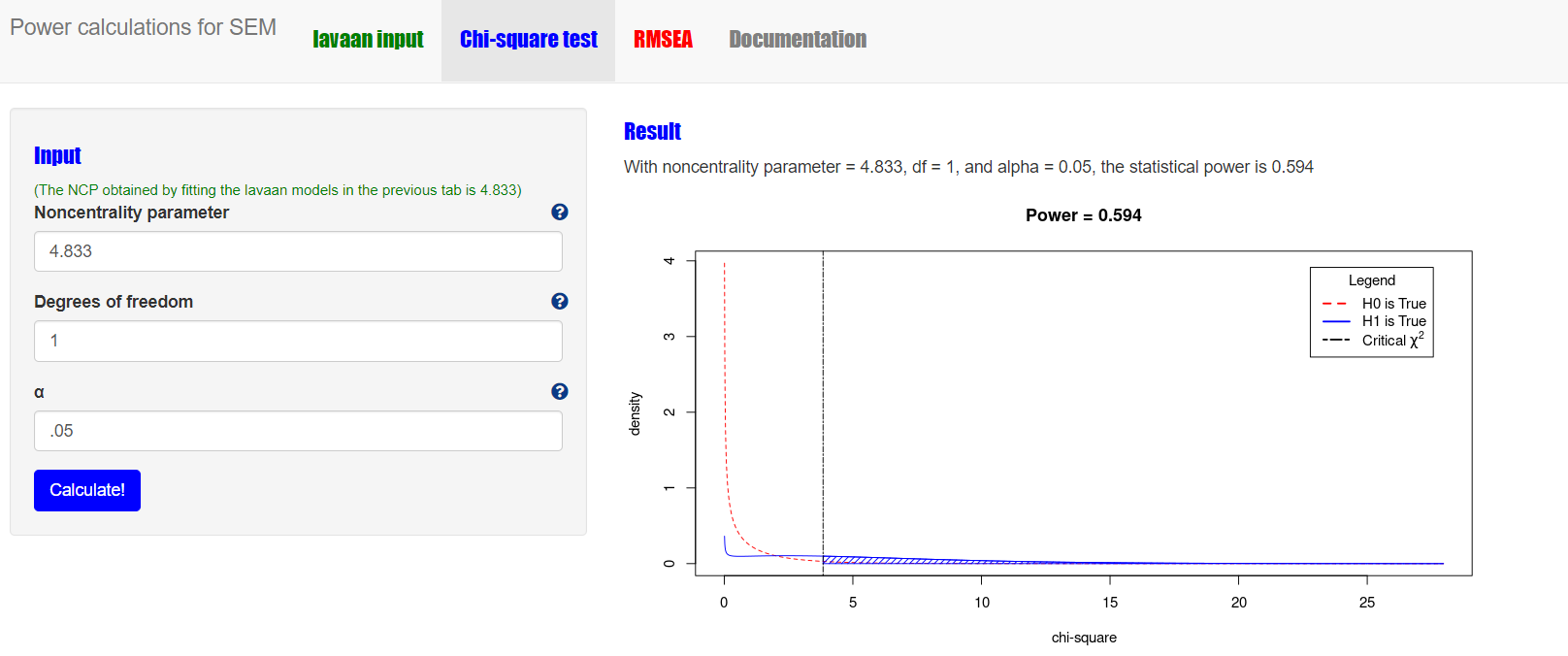


**5. Insert NCP value**

**I**

**6. Insert Df = 1**

**I**

**8. Click “Calculate!”**

**I**

**7. Insert alpha = .05**

**I**

**Model 3C: H_1_ that includes one medium-sized recalibration effect**

### Model H1.C: Model including one reprioritization effect

# factor loadings

PHYS_baseline =~ .5*PF_baseline + .5*RP_baseline + .5*BP_baseline +

.5*GH_baseline

MENT_baseline =~ .5*VT_baseline + .5*SF_baseline + .5*RE_baseline +

.5*MH_baseline

PHYS_followup =~ .5*PF_followup + .5*RP_followup + .5*BP_followup +

.5*GH_followup

MENT_followup =~ .5*VT_followup + .5*SF_followup + .5*RE_followup +

.5*MH_followup

# (co)variances underlying latent factors

PHYS_baseline ~~ 1*PHYS_baseline + .5*MENT_baseline + .5*PHYS_followup +

.3*MENT_followup

MENT_baseline ~~ 1*MENT_baseline + .3*PHYS_followup + .5*MENT_followup

PHYS_followup ~~ 1*PHYS_followup + .5*MENT_followup

MENT_followup ~~ 1*MENT_followup

# residual (co)variances

PF_baseline ~~ .75*PF_baseline + .1*PF_followup

RP_baseline ~~ .75*RP_baseline + .1*RP_followup

BP_baseline ~~ .75*BP_baseline + .1*BP_followup

GH_baseline ~~ .75*GH_baseline + .1*GH_followup

VT_baseline ~~ .75*VT_baseline + .1*VT_followup

SF_baseline ~~ .75*SF_baseline + .1*SF_followup

RE_baseline ~~ .75*RE_baseline + .1*RE_followup

MH_baseline ~~ .75*MH_baseline + .1*MH_followup

PF_followup ~~ .75*PF_followup

RP_followup ~~ .75*RP_followup

BP_followup ~~ .75*BP_followup

GH_followup ~~ .75*GH_followup

VT_followup ~~ .75*VT_followup

SF_followup ~~ .75*SF_followup

RE_followup ~~ .75*RE_followup

MH_followup ~~ .75*MH_followup

# intercept values

PF_baseline ~ 0*1

RP_baseline ~ 0*1

BP_baseline ~ 0*1

GH_baseline ~ 0*1

VT_baseline ~ 0*1

SF_baseline ~ 0*1

RE_baseline ~ 0*1

MH_baseline ~ 0*1

PF_followup ~ 0*1

RP_followup ~ 0*1

BP_followup ~ 0*1

GH_followup ~ 0*1

VT_followup ~ 0*1

SF_followup ~ 0*1

RE_followup ~ 0*1

MH_followup ~ 0.5*1

# underlying latent factor means

PHYS_baseline ~ 0*1

MENT_baseline ~ 0*1

PHYS_followup ~ 0.5*1

MENT_followup ~ 0.5*1

**Calculate statistical power of the chi-square difference test for Step 3 with power4SEM**

**1. Use the “lavaan input” page**


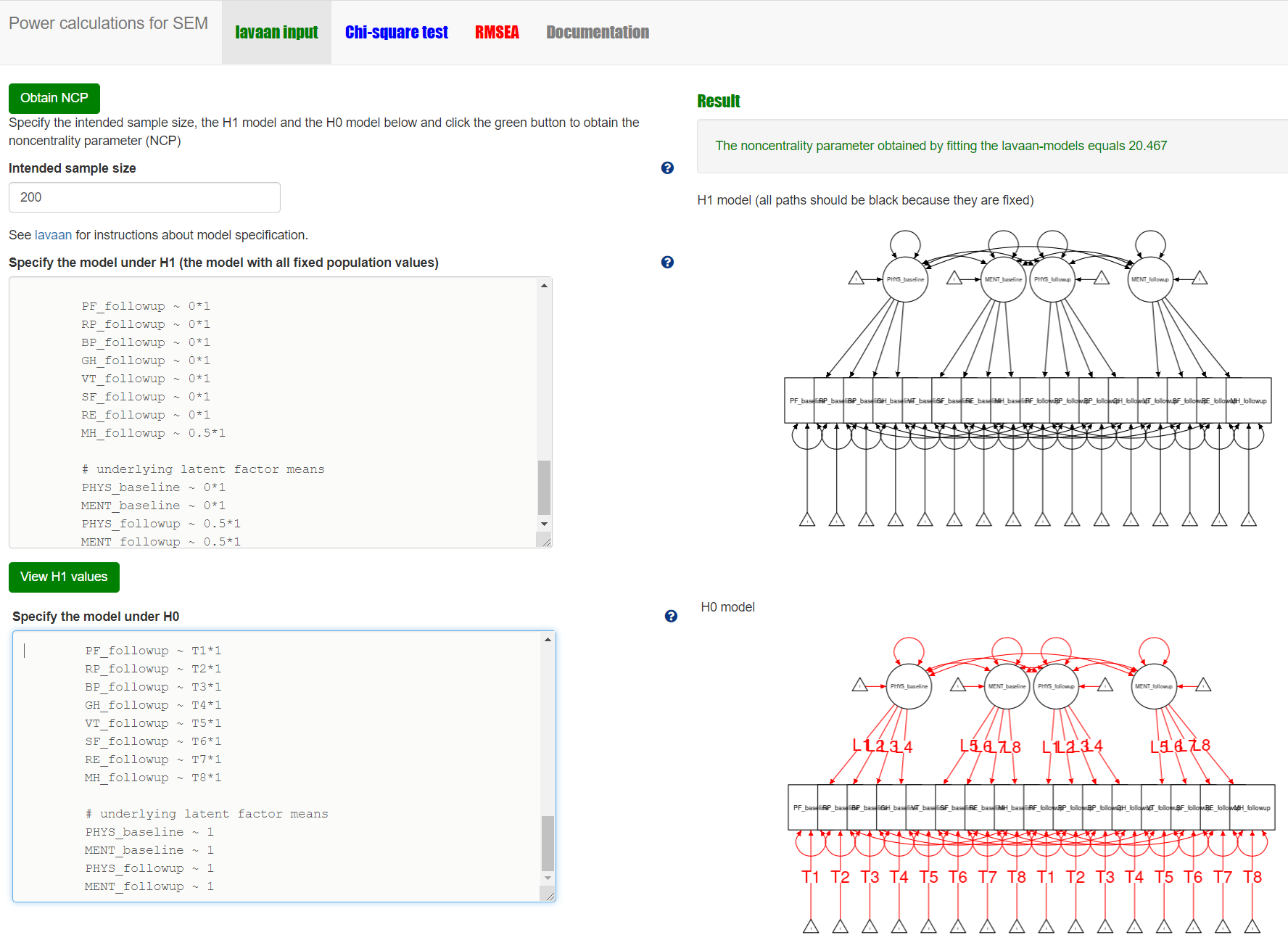


**3. Use N=200 and click “Obtain NCP”**

**2. Insert H_0_ and H_1_ syntax**

**4. Go to the “Chi-square test” page**


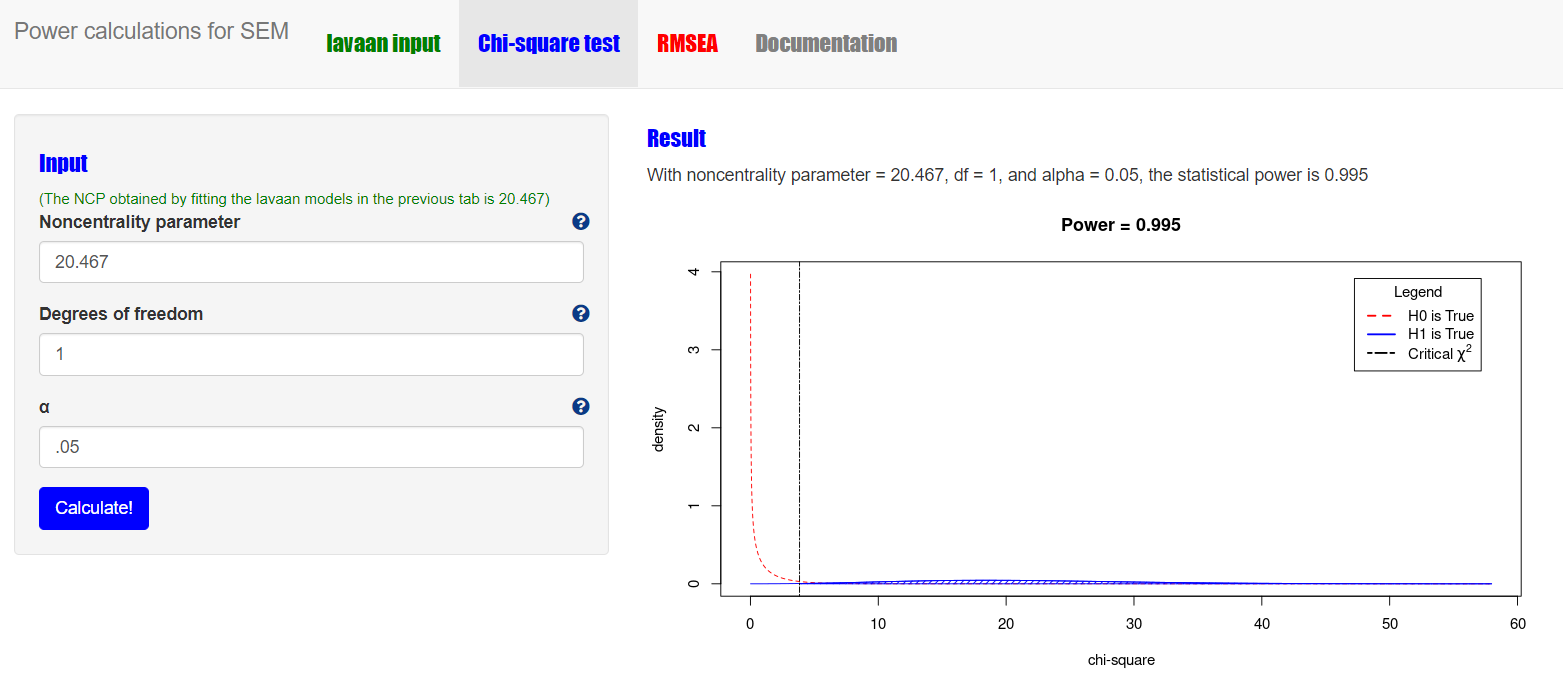


**5. Insert NCP value**

**I**

**6. Insert Df = 1**

**I**

**8. Click “Calculate!”**

**I**

**7. Insert alpha = .05**

**I**
